# CRISPR/Cas System and Factors Affecting Its Precision and Efficiency

**DOI:** 10.3389/fcell.2021.761709

**Published:** 2021-11-24

**Authors:** Nasir Javaid, Sangdun Choi

**Affiliations:** ^1^ Department of Molecular Science and Technology, Ajou University, Suwon, South Korea; ^2^ S&K Therapeutics, Ajou University Campus Plaza, Suwon, South Korea

**Keywords:** CRISPR/cas system, classification, host DNA repair, genome-editing efficiency, epigenome

## Abstract

The diverse applications of genetically modified cells and organisms require more precise and efficient genome-editing tool such as clustered regularly interspaced short palindromic repeats/CRISPR-associated protein (CRISPR/Cas). The CRISPR/Cas system was originally discovered in bacteria as a part of adaptive-immune system with multiple types. Its engineered versions involve multiple host DNA-repair pathways in order to perform genome editing in host cells. However, it is still challenging to get maximum genome-editing efficiency with fewer or no off-targets. Here, we focused on factors affecting the genome-editing efficiency and precision of CRISPR/Cas system along with its defense-mechanism, orthologues, and applications.

## 1 Introduction

The manipulation of DNA molecules to study genes and their applications in the field of biotechnology became possible through the development of recombinant DNA technology in the 1970s. With advancements in genome engineering, it has become possible to edit the target genome at the systematic level under natural cellular conditions. The function of specific genes or regulatory elements can be studied by insertion, deletion, or modification of the associated DNA sequences. The larger scale network of genes or proteins can be interrogated by multiplex genome editing which helps in the understanding of complex polygenic disorders. The cellular organization and architecture of the genomic material and its associated functions are revealed by manipulating chromatin and transcriptional regulation. The precise manipulation enables reconstruction of biological systems with enhanced or better features, e.g., genetically modified microbes, animals, and plants. This targeted modification can be used in human gene therapy to correct harmful genetic mutations. The successful execution of these processes holds immense promise to transform various fields, such as medicine, biotechnology, and basic science.

It is difficult to manipulate billions of DNA bases in the eukaryotic genome. The first breakthrough came in terms of homologous recombination (HR) based transgene integration at the target site, but efficiency was quite low (1 in 10^6^–10^9^ cells) (Capecchi, 1989). This process was significantly increased by introducing site-specific DNA double-strand breaks (DSBs) (Rudin et al., 1989; Bibikova et al., 2001). However, in the absence of a repair template, DSBs result in insertion and deletion mutations (indels) via the error-prone DNA repair pathway non-homologous end joining (NHEJ) (Bibikova et al., 2002). In this regard, four classes of programmable DNA-binding proteins have been engineered, including meganucleases from microbial mobile genetic elements (Smith et al., 2006), zinc finger nucleases (ZFNs) (Urnov et al., 2005), transcription activator-like effector nucleases (TALENs) (Moscou and Bogdanove, 2009; Miller et al., 2010), and the RNA-guided DNA endonuclease Cas (Jinek et al., 2012a; Gasiunas et al., 2012). Of these, the Cas enzyme derived from the microbial adaptive immune system CRISPR (clustered regularly interspaced short palindromic repeats) is considered the most efficient, advanced, and user-friendly.

The CRISPR/Cas system can be used to target any part of the human genome associated with protospacer adjacent motif (PAM) sequence by using short guide RNA, which follows Watson–Crick base pairing to recognize the target sequence. The CRISPR story began in 1987 when a set of 29 nucleotides was found downstream of the *iap* gene in *Escherichia coli*, a product which caused the conversion of alkaline phosphatase (Ishino et al., 1987). There were five 32 nucleotide long non-repetitive sequences intervening in these 29 nucleotide repeats, a phenomenon opposite to most repetitive elements that usually take the shape of tandem repeats like those of TALE repeat monomers. During the next 10 years, genome sequencing of various bacterial and archaeal strains confirmed the presence of additional such repeat elements (Mojica et al., 2000), which were eventually named CRISPR (Jansen et al., 2002). In addition, various well-conserved CRISPR-associated (cas) gene clusters were discovered adjacent to the repeat elements that led to initial classification of the CRISPR system into three main types (types I–III) (Jansen et al., 2002; Haft et al., 2005; Makarova et al., 2011). In types I and III of the CRISPR system, multiple Cas proteins recognize and destroy target nucleic acids, while type II consists of lesser number of these proteins (Brouns et al., 2008; Hale et al., 2009).

**FIGURE 1:**
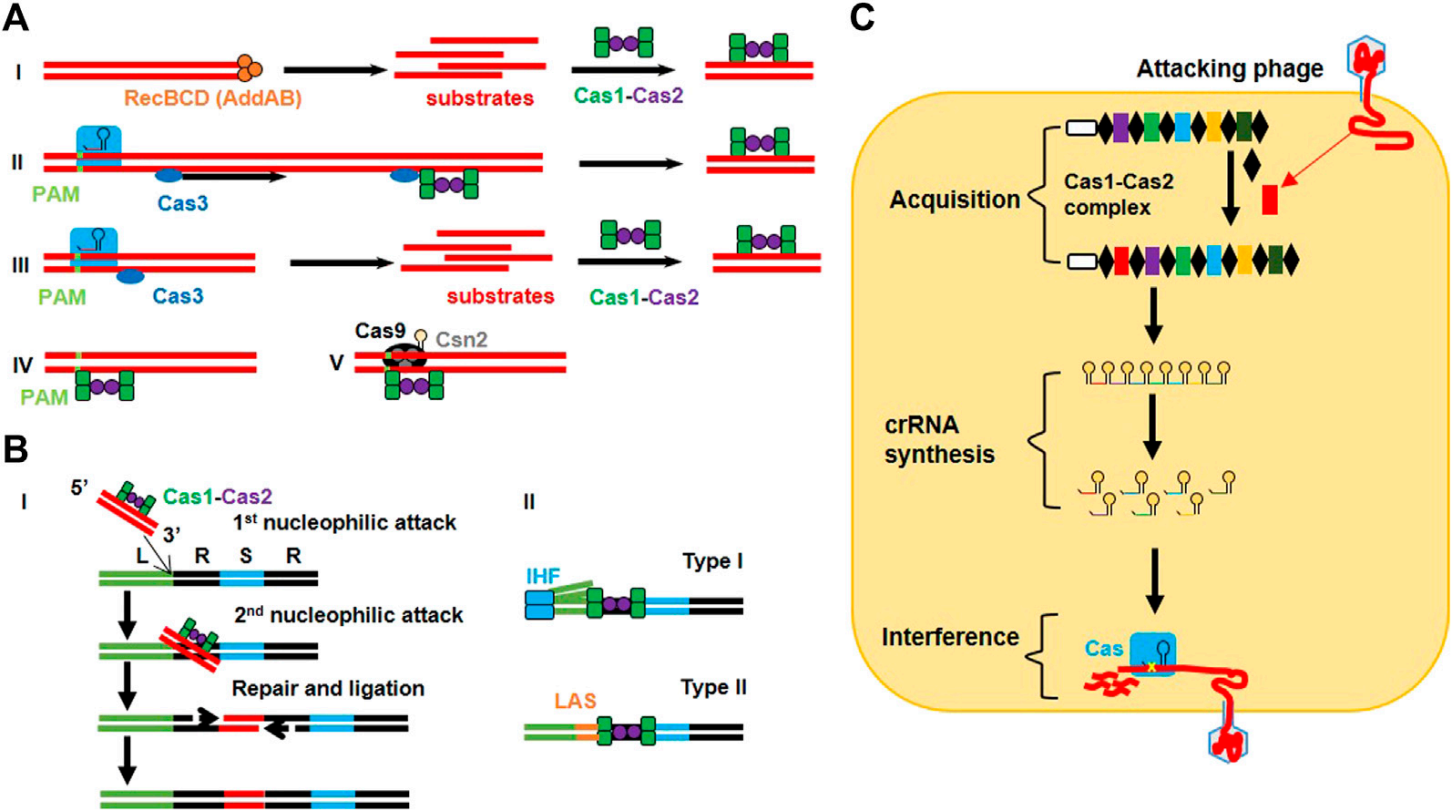
Detailed mechanism of CRISPR immunity in bacteria. **(A)** Protospacer acquisition: (I) Type I naive adaptation involves nuclease/helicase RecBCD in Gram-negative organisms (or AddAB in Gram-positive organisms) to generate substrate products for the Cas1-Cas2 complex. (II) Type I nuclease-inactive Cas3 (inCas3)-primed mechanism involves a conformational change in the Cse1 subunit of Cascade after imperfect recognition of the target region that recruits inCas3. The Cas3 moves along the target strand to find the suitable site where it recruits the Cas1-Cas2 complex for spacer acquisition. (III) The type I Cas3 mechanism allows Cascade to recognize foreign DNA in a PAM-dependent manner and to recruit Cas3 to generate substrate products for the Cas1-Cas2 complex. (IV) Type I Cas3-independent mechanism employs the inherent ability of the Cas1-Cas2 complex to recognize the target in a PAM-dependent manner. (V) Type II adaptation utilizes the PAM-interacting domain (PID) of Cas9 (loaded with tracrRNA) to guide the Cas1-Cas2 complex (along with accessory protein Csn2) for retrieving protospacers. **(B)** Spacer integration into the CRISPR array: (I) The Cas1-Cas2 complex guides the protospacer (3′-OH) to execute 1st nucleophilic attack at the leader end (L) of the first repeat (R). This bends the repeat DNA to help protospacer in executing 2nd nucleophilic attack at the spacer end (S) of the same repeat. As a result of these cleavage-ligation reactions, a double-stranded protospacer is bound to single-stranded repeat sequences *via* its 3′ ends. The gaps are filled and repaired via polymerases and ligases. (II) The type I system allows integration host factor (IHF) to bend DNA after binding to the conserved sequence in the leader region, which allows the Cas1-Cas2 complex to interact with the leader and IHF to perform the cleavage-ligation reaction. In the type II system, recognition of the leader anchoring sequence (LAS) by Cas1 is sufficient to execute polarized integration of the spacer. **(C)** Sequential phage interference: CRISPR immunity consists of three steps. The acquisition step involves integration of the new spacer (red) between the two repeat elements (black) with the help of the Cas1-Cas2 complex. crRNA synthesis step involves the transcription of the CRISPR array from the leader sequence (white) to make pre-crRNA which is further converted into a series of crRNAs. During the interference step, crRNA is assembled with the Cas protein (blue) to make the effector complex that targets and cleaves the complementary sequence in the genome of the attacking phage.

In 2005, phage-associated and extrachromosomal origins of the intervening spacer sequences between the successive direct repeats were confirmed through a systematic analysis (Bolotin et al., 2005; Mojica et al., 2005; Pourcel et al., 2005). Further studies demonstrated that CRISPR loci can be transcribed (Tang et al., 2002) and those viruses cannot infect the host cell which have their relevant spacer sequence integrated into the host genome (detailed mechanisms are shown in Figure 1) (Mojica et al., 2005). These studies speculated the immune memory and defensive nature of CRISPR arrays against invading bacteriophages (Mojica et al., 2005; Pourcel et al., 2005). The immune nature of CRISPR loci proposed a challenging puzzle regarding the working mechanism of spacers which led to several hypotheses, including that spacers cleave target DNA at Watson–Crick base pairing region by directing Cas enzymes (Bolotin et al., 2005) or that spacers behave like RNA guides to cleave viral transcripts in a mechanism similar to that of RNAi (Makarova et al., 2006).

The first experimental proof for a natural, nucleic acid based-adaptive immune role of the type II CRISPR system was revealed while working with *Streptococcus thermophiles,* a bacterial strain used in the dairy industry (Barrangou et al., 2007). A series of studies revealed the functional mechanism of adaptive immunity conferred by all three types of CRISPR loci (Figure 1). In the type I CRISPR system of *E. coli*, spacers containing small crRNAs are produced by transcription of CRISPR arrays in which the spacer region guides the Cas protein for its nuclease activity (Brouns et al., 2008). In the type III-A CRISPR system of *Staphylococcus epidermidis*, Cas enzymes block plasmid conjugation by targeting DNA rather than RNA (Marraffini and Sontheimer, 2008). However, a different type III system (type III-B) in *Pyrococcus furiosus* also revealed the RNA cleaving ability of crRNA (Hale et al., 2009; Hale et al., 2012). The importance of PAMs was revealed by analyzing the circumvention in CRISPR interference because of a mutation in the PAM region (Bolotin et al., 2005; Deveau et al., 2008). However, the type III system requires mismatches between the target DNA and the 5′ end of crRNA for plasmid interference (Marraffini and Sontheimer, 2010).

**FIGURE 2:**
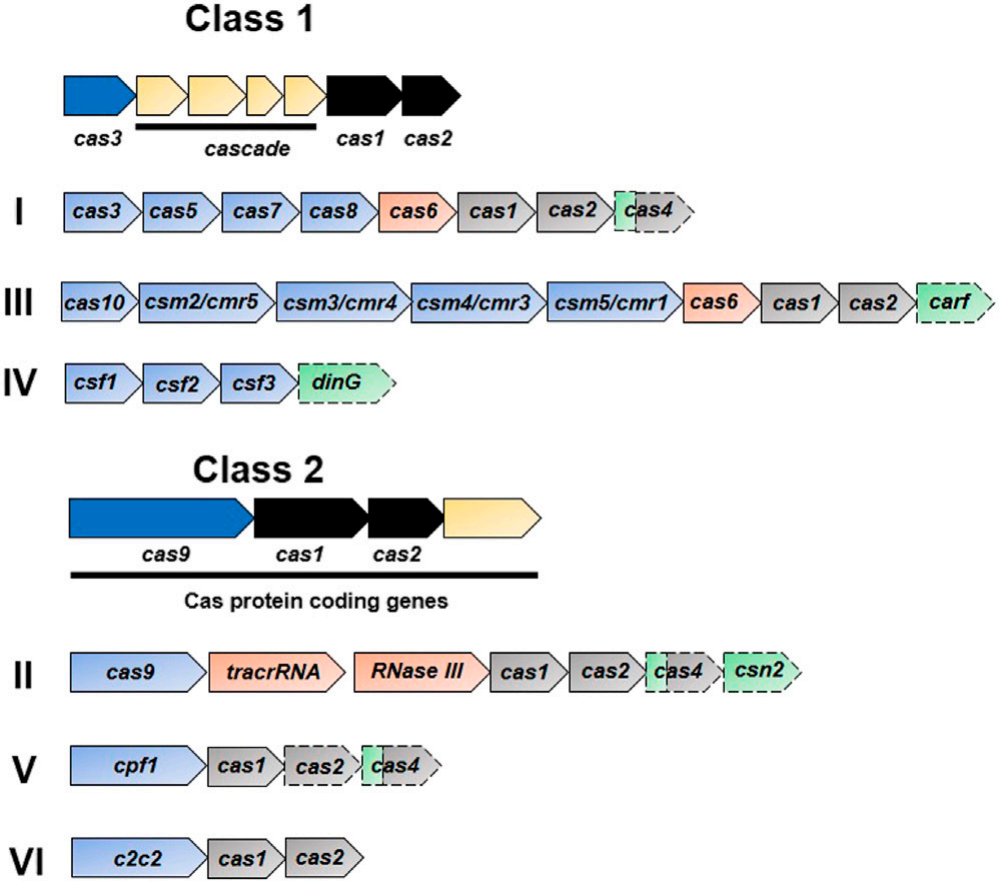
Functional organization of CRISPR systems. The CRISPR/Cas system has two main classes depending upon the nature of the effector nuclease complexes: class 1 has a multi-protein complex while there is single main protein in class 2. Each class is further subdivided into three main types based upon the signature and complementary genes. Representative operons specific to each type are shown in the figure. Dispensable elements are represented by a dashed outline, while the two colors for cas4 indicate the involvement of gene product in two stages. Genes involved in the interference, crRNA-synthesis, adaptation, and accessory role are represented in blue, orange, black, and green, respectively.

Until now, the CRISPR/Cas system has been classified into six types (type I–VI) based on their signature genes which are grouped into two main classes depending upon the nature of the effector complexes (Figure 2) (Makarova et al., 2011; Makarova et al., 2015; Shmakov et al., 2015). The types included in the class I system (types I, III, and IV) are composed of effector complexes with multiple subunits while those included in the class II system (II, V, and VI) are composed of effector complexes with a single subunit (Makarova et al., 2015; Shmakov et al., 2015). The first discovered and most studied types include types I–III (as mentioned above), while types IV–VI were discovered afterwards (Makarova et al., 2015; Makarova and Koonin, 2015; Shmakov et al., 2015). In the type I system, Cas3 is the signature protein that cleaves the target DNA, recognized by the multiprotein-crRNA complex Cascade (CRISPR associated complex for antiviral defense), with the help of its helicase and nuclease domains. The type II system uses its signature protein, Cas9, for interference. The type III system assembles its signature protein, Cas10, into a cascade-like interfering complex to find and destroy the target. The uncharacterized protein of the type IV system, Csf1, is suggested to be the part of the cascade-like complex, however these systems often exist alone as *cas* genes without any allied CRISPR array (Makarova and Koonin, 2015). A single Cas9-like nuclease in the type V system might be either Cpf1, C2c1, or C2c3 depending upon the subtype (Zetsche et al., 2015a; Shmakov et al., 2015). The type VI system has a single large protein, C2c2, with two HEPN (higher eukaryotes and prokaryotes nucleotide binding) RNase domains (Shmakov et al., 2015).

## 2 Era of Genome Editing

Application of the CRISPR tool in genome editing began after discovery of the basic components of the native type II CRISPR system. Cleavage of the target DNA in *S. thermophiles* is mediated by only the Cas9 enzyme among all members of the *cas* gene cluster (Figure 2) (Garneau et al., 2010). Later, noncoding *trans*-activating crRNA (tracrRNA) was discovered as a key component involved in generating and processing the crRNA, which facilitates RNA-guided targeting of the Cas9 enzyme after hybridization with the crRNA (Deltcheva et al., 2011). This hybrid of tracrRNA and crRNA combines with Cas9 and endogenously expressed RNase III to process transcripts of the CRISPR array into mature crRNA (Deltcheva et al., 2011). These studies revealed the minimum essential components (Cas9, crRNA, and tracrRNA) required to work in the type II CRISPR nuclease system. Because of the genome editing ability of ZFNs and TALENs, Cas9 endonuclease was also thought to be exploited in the same way which started a new race afterwards.

In 2011, it was revealed that the type II CRISPR locus derived from the *Streptococcus thermophilus* is able to perform CRISPR interference in *Escherichia coli* demonstrating transferability of this technology (Sapranauskas et al., 2011). The biochemical characterization of the Cas9 purified from *Streptococcus pyogenes* and *S. thermophilus* revealed that this enzyme cleaves target DNA after being guided by the crRNAs (Gasiunas et al., 2012). Moreover, *in vitro* cleavage of the target DNA is performed by a single guide RNA (sgRNA) which is formed by fusing the target DNA-specific crRNA and tracrRNA (Figure 2) (Jinek et al., 2012a).

**FIGURE 3:**
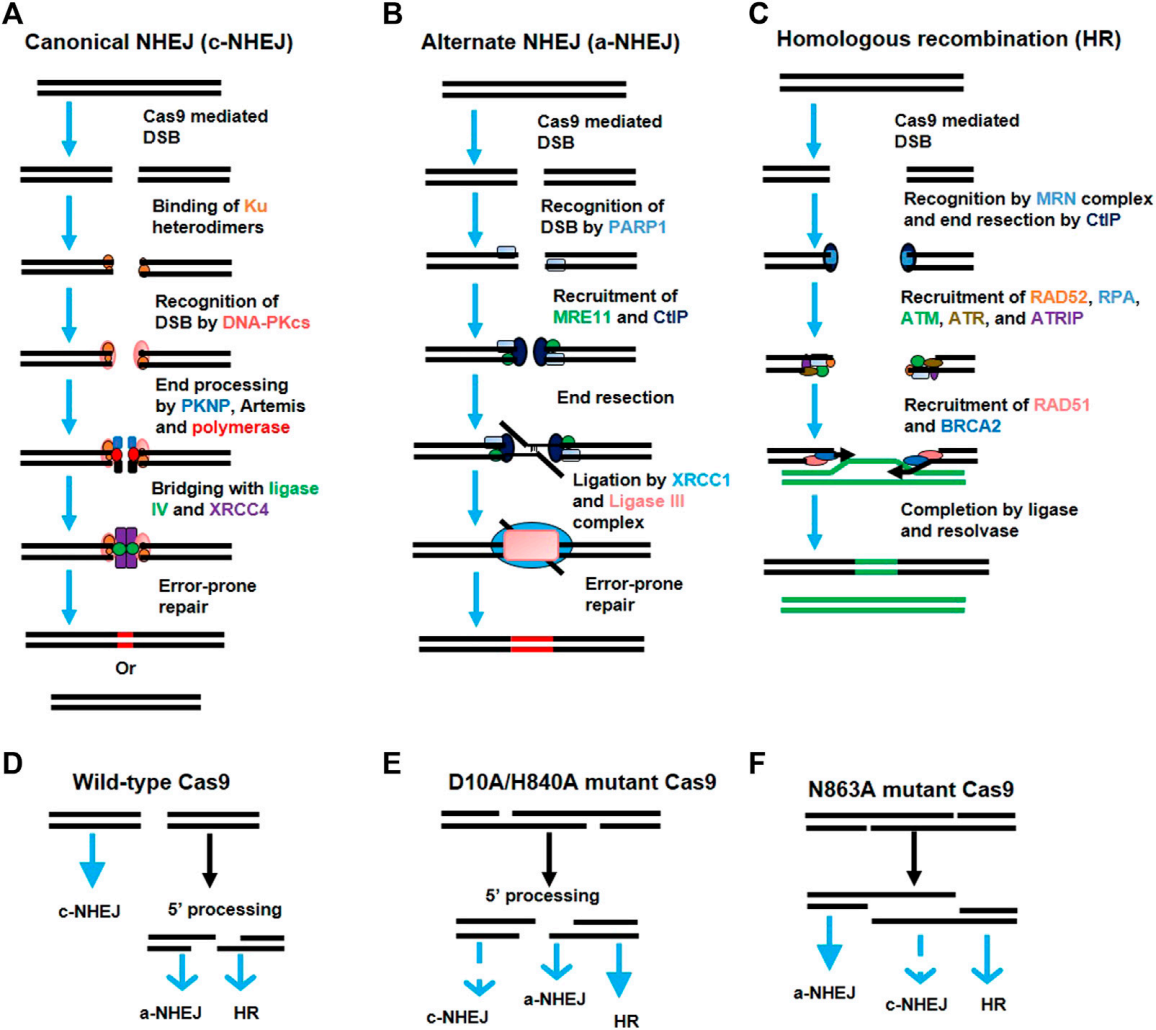
Activation of various DNA repair pathways after generation of double-strand breaks (DSBs) by Cas9. **(A)** The canonical non-homologous end joining (c-NHEJ) pathway. Broken DNA ends are recognized by the Ku heterodimer (Ku70 and Ku80), which recruits the DNA-PK catalytic subunit (DNA-PKcs). DNA-PKcs recruits various proteins like PKNP, Artemis, and polymerase for end processing. The ends are rejoined by ligase IV and XRCC4 with error-prone repair. **(B)** Alternate non-homologous end joining (a-NHEJ) pathway. The DSBs are recognized by the PARP1 protein which recruits MRE11 and CtlP for end resection. The internal microhomologies are associated with the larger deletions at junctions than that of c-NHEJ. Ligase III and XRCC1 ligate the strand ends. **(C)** The homologous recombination (HR) pathway. The DSBs are recognized by the MRE11-RAD50-NBS1 (MRN) complex which activates the DNA damage response *via* ATM kinase. The single-strand DNA (ssDNA) is formed after 5′ to 3′ end resection by CtlP which allows RAD52 and DNA replication protein A (RPA) to recognize the exposed ssDNA. This activates the Ataxia Telangiectasia and Rad3-related protein (ATR) to assist in HR repair. The RPA-coated ssDNA is replaced by the BRCA2 and RAD51 protein which performs strand invasion via searching for the homologous sequence. The junction is resolved and the ends are joined *via* resolvase and ligase. **(D–F)** Choice of DNA-repair pathway after DSBs. The predominance of a particular DNA-repair pathway (bold blue arrow) depends upon the type of lesion generated by a particular variant of Cas9.

By 2013, two simultaneous studies revealed the successful engineering of a type II system derived from *S. pyogenes* (Cong et al., 2013a; Mali et al., 2013b) and *S. thermophiles* (Cong et al., 2013a) to perform genome editing in mammalian cells. Homology directed repair (HDR) or NHEJ-mediated genome editing is stimulated in the mammalian cell genome after Cas9-mediated cleavage (details in Figure 3); this cleavage is directed by the heterologous expression of sgRNA (Cong et al., 2013a; Mali et al., 2013b) as well as a mature hybrid of crRNA-tracrRNA (Cong et al., 2013a). This system can also be used to target various genes simultaneously using multiple guide RNAs. Since then, the CRISPR/Cas9 system has been applied to various experimental models for genome editing by multiple laboratories (Sander and Joung, 2014).

By keeping the necessity of each of the three components (Cas9, crRNA, and tracrRNA) under consideration, researchers have reduced it to two components via making sgRNA (crRNA + tracrRNA). This conversion has made this tool more user-friendly for transcriptional control, genome editing, imaging, and RNA targeting. It has allowed it to be used in various types of cells and organisms ranging from stem cells and primary human T-cells to bacteria, fungi, plant, mice, and monkeys (Jiang and Marraffini, 2015; Sternberg and Doudna, 2015; Lin et al., 2017; Li et al., 2018). Cas9 has been used to produce various light-and chemical-inducible constructs for better spatiotemporal control as well as to employ orthologues of smaller sizes and different PAMs for easier packaging in adeno-associated virus vectors and broader targeting, respectively (Nihongaki et al., 2015a; Zetsche et al., 2015b; Davis et al., 2015; Polstein and Gersbach, 2015; Ran et al., 2015; Havlicek et al., 2017; Edraki et al., 2018; Shao et al., 2018).

Although other interference complexes have the potential to be used for genome manipulation, their multiple-subunit cascade composition makes them less suitable for genome editing unlike Cas9. However, their ability to bind stably has been employed for transcriptional silencing in *E. coli* (Rath et al., 2014). The *Pyrococcus furiosus* and *Sulfolobus solfataricus* derived Cmr system has been engineered to target various RNA substrates; however, targeting in mammalian cells has not been reported (Hale et al., 2009; Hale et al., 2012; Hale et al., 2014; Li et al., 2015a; Li et al., 2017). Three Cpf1 homologs have been validated in various cells for genome editing (Zetsche et al., 2015a; Kim et al., 2016; Zhang et al., 2017; Świat et al., 2017). The recognition of PAM different from those of Cas9 and the generation of staggered ends after cutting may facilitate the application of Cpf1 for genome editing by involving different DNA repair pathways. However, further investigation is needed to validate the off-targets and efficiency. The properties of various orthologues and engineered enzymes associated with CRISPR/Cas system are listed in Supplementary Table S1.

**FIGURE 4:**
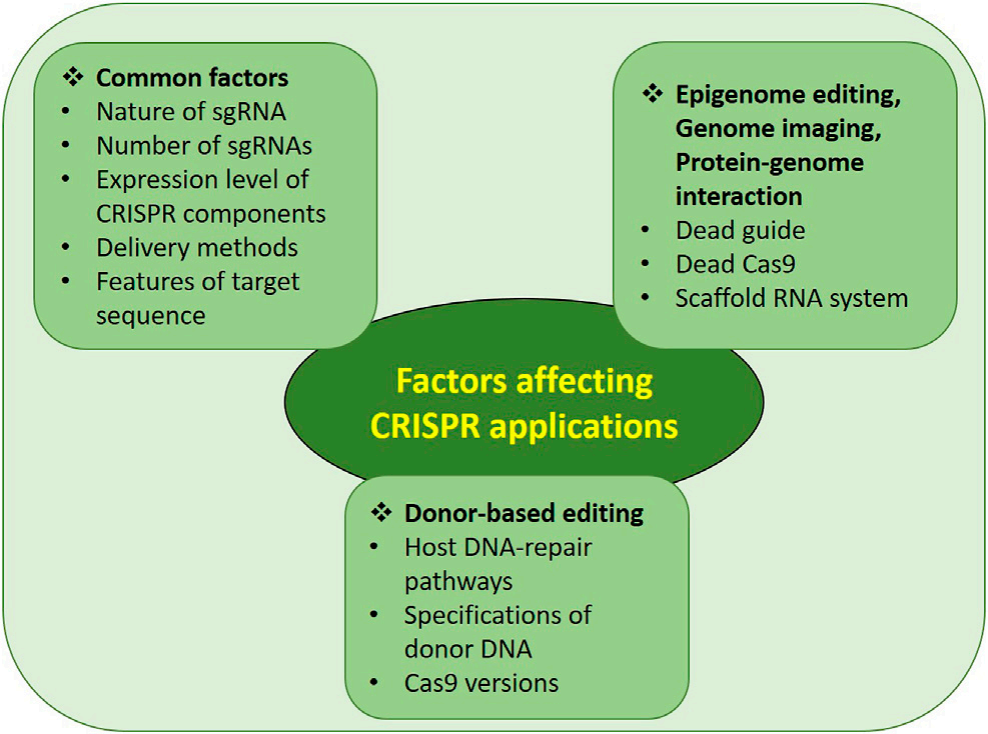
Factors affecting efficiency and specificity of CRISPR/Cas system.

The genome editing process mediated by the CRISPR/Cas system depends upon various factors. Until now, many different ways have been adopted to increase genome-editing efficiency with minimized off-targets. This review describes such practical improvements so that researchers can choose the best conditions to achieve maximum on-target efficiency. The factors affecting various CRISPR applications are summarized in Figure 4 with the details mentioned below.

## 3 Factors Affecting Most CRISPR Applications

### 3.1 Nature of sgRNA

#### 3.1.1 Origin and GC Content

Genome editing efficiency using spacers varies depending upon the genomic region from where they were derived. Editing is highest for promoter regions followed by the exonic regions and intronic regions. This is because of the variability in retrieving spacers to the relevant target region, which further depends upon the variable GC content of the spacers (Labuhn et al., 2017), as different regions of the genome adopt various conformations based upon variation in their GC content (Pozzoli et al., 2008; Amit et al., 2012).

The GC content of the spacer region of designed sgRNA indirectly indicates the strength of the interaction between the spacer and protospacer sequences in various life domains that may affect the overall endonuclease activity of various Cas enzymes. In animals, spacers with average GC content tend to be more effective unlike those with unusually low or high GC content (Wang et al., 2014). Similarly, spacers in plants with GC content between 30 and 80% have been practically validated (Liang et al., 2016). However, lesser editing efficiency is observed in plants with 40% GC content as compared to those with higher GC content (Pan et al., 2016b). In mammalian cells, spacers with very low or very high GC content are less effective; however, 40–60% content is favorable for efficient genome editing (Doench et al., 2014; Wang et al., 2014; Liu et al., 2016c). Overall, spacers with more than 50% GC content often show high genome editing efficiency in microbes, animals, and plants (Jiang et al., 2013a; Jiang et al., 2013b; Feng et al., 2014; Wang et al., 2014; Zhang et al., 2014; Pan et al., 2016a). Inefficient sgRNAs can also be excluded by monitoring the GC content in the PAM distal region (4–13 nucleotides) (Labuhn et al., 2017). However, bulges arise between the sgRNA-DNA hybrid when GC content equals 70% (Lin et al., 2014c). DNA bulges formed due to mismatches 7–10 bp from PAM; thus, the 3′end or 5′end can result in mismatches, so these must be avoided (Lin et al., 2014b).

#### 3.1.2 Nucleotide Preferences and Alterations

Nucleotide composition (purine or pyrimidine) of spacers may also affect Cas9 binding and nuclease activity. In animal models, it has been reported that the 3′ sequence of the spacer contributes to Cas9 binding preference, and purines are better to recruit the Cas9 enzyme than pyrimidines which eventually affects its nuclease activity (Wang et al., 2014). Doench et al., 2014) reported Cas9 preference for guanine and against cytosine at position 20; for cytosine and against guanine at position 16; for adenine in the middle and against cytosine at position three of the spacer sequence. Similarly, Xu et al. (2015a) revealed Cas9 preference for guanine at positions 19 and 20; against thymine from positions 17 to 20; for cytosine at position 18 (CRISPR/Cas9 complex DNA cleavage site); for adenines from positions nine to sixteen; and for guanines from positions four to seven. Moreover, PAM downstream nucleotides, unlike spacer upstream sequences, contribute to the editing efficiency of spacer (Doench et al., 2014; Xu et al., 2015a). The sequences rich in guanine can form noncanonical stable structures *in vivo*, called as G-quadruplexes (Huppert, 2008). Moreno-Mateos et al. (2015) injected *in vitro* transcribed guide sequences into zebrafish model and reported that sequences with more than eight guanines undergo G-quadruplex structures which are more efficient to edit due to their increased stability. However, this modification is not essential in case the guide is being expressed from constitutive promoter (like U6) after lentiviral transduction. On the other hand, these G-rich guides are not efficient in cultured mammalian cells (Malina et al., 2015). In contrast, no such nucleotide preference is observed in plants revealing a major difference in spacer designed to use in plants and animals (Liang et al., 2016).

Transcription from the U6 promoter by RNA polymerase III is inhibited when there is TTT stretch in the DNA, so guide RNAs with a UUU stretch (particularly in the seed region) are inefficient under the U6 promoter, and editing is more favorable when a stable duplex is formed between the target DNA and the guide RNA (Wong et al., 2015). Moreover, particular sequence motifs that affect the synthesis of the tracrRNA structures required for Cas9 interaction may also decrease guide activity. The presence of uridine at four nucleotide proximal to PAM makes it difficult for guide RNA to interact with Cas9 eventually resulting in lower activity (Wang et al., 2014; Hart et al., 2015).

To optimize sgRNA structure, researchers found that 1) substituting one of the T’s in four consecutive T’s with an A (to distort the string) shortly downstream of the spacer and 2) extending the sgRNA duplex region by five-nucleotides changes the sgRNA transcription rate and its structure, respectively, resulting in improved efficiency (Chen et al., 2013; Dang et al., 2015). This strategy also produced promising results with SaCas9 sgRNAs, signifying that this could be applied to other Cas9 orthologues to achieve maximum efficiency for different applications (Chen et al., 2016; Tabebordbar et al., 2016).

#### 3.1.3 Secondary Structure

Ma et al. (2013) targeted the PVALB gene of humans with various sgRNAs and concluded that the absence of sgRNA secondary structures increases binding with the target gene, leading to more efficient genome editing. However, subsequent studies supported the existence of a secondary structure. Briefly, sgRNA consists of a crRNA sequence {guide/spacer [20 nucleotides (nt)] + repeat region [12 nt]} and a tracrRNA sequence [anti-repeat (14 nt)] and three stem loops (stem loops 1, 2, and 3). The fourth loop (called the RAR loop) is formed by the bases of repeat and anti-repeat regions that trigger RNase III-mediated processing of precursor CRISPR RNA (pre-crRNA). Stem loop 1 is important for formation of the Cas9-sgRNA-DNA functional complex while the other two loops provide stability to the complex and enhance *in vivo* function (Nishimasu et al., 2014). Liang et al. (2016) reported that all stem loops (except stem loop 1) must have an intact secondary structure for efficient genome editing.

#### 3.1.4 Modified Versions

GGX20 modification of sgRNA involves addition of two additional mismatched guanines at the 5′ end of the designed guide RNA. Several studies have shown that this sgRNA modification enables them to differentiate the on-target site from other homologous sites differing by two or more nucleotides eventually increasing on-target specificity of sgRNA 10–100 times, but it also affects editing efficiency at the on-target and off-target locations (Cho et al., 2013; Fu et al., 2013; Cho et al., 2014; Sung et al., 2014; Kim et al., 2015). Moreover, only certain guides are compatible with such a modification, so there is a need to further validate this modification with different lengths and chemically modified sgRNAs.

Another specificity mediator of CRISPR/cas9 technology is the length of the designed sgRNA. Extending Spcas9 guide sequence from 20 nt to 30 nt to increase specificity results in its processing back to natural 20 nt length, so it is useless to increase length (Ran et al., 2013a). Short length (17 or 18 nt) spacer sequences, called truncated spacers or guides, have been reported to potentially increase Cas9 binding sensitivity to mismatches present within a smaller complementary sequence which causes more accurate but less active genome editing (Pattanayak et al., 2013; Fu et al., 2014). Even after eliminating off-target activity at many sites, truncated guides produce new off-target sites due to their shorter length (Fu et al., 2014; Tsai et al., 2015; Wyvekens et al., 2015; Slaymaker et al., 2016). Interestingly, these truncated guides show very low on-target efficiency when used with modified SpCas9 versions, such as eSpCas9 and SpCas9-HF (Kleinstiver et al., 2016; Slaymaker et al., 2016). Improvements in specificity need to be evaluated in other Cas9 orthologues with intact editing efficiency using these guide modifications (Hou et al., 2013; Friedland et al., 2015; Ran et al., 2015). Jinek et al. (2012b) detected efficient *in vitro* cleavage using truncated crRNA and tracrRNA but failed to detect cleavage at various loci previously modified using crRNA-tracrRNA duplexes with identical guide sequences (Cong et al., 2013b). Another study revealed that this cleavage difference is due to the length of tracrRNA sequence, as they used sgRNAs with tracrRNA tails extended to +67 and +85 nt to mediate cleavage at all previously tested target sites and found a five-fold increase in the level of indels than in the corresponding crRNA-tracrRNA duplex. They also observed increased expression of sgRNAs having longer tracrRNA sequences, predicting that higher sgRNA stability or expression is responsible for improved cleavage of the target sequence (Ran et al., 2013a).

In one study, full length 100 nt sgRNAs (crRNA + tracrRNA) were synthesized with three various chemical modifications of 2′-O-methyl (M), 2′-O-methyl 3′phosphorothioate (MS), or 2′-O-methyl 3′thioPACE (MSP) at the 5 and 3′ termini, which improved editing efficiency in CD34 ^+^ hematopoietic progenitor and stem cells, and human primary T-cells along with off-target activity at a few sites (Hendel et al., 2015). In another study, a 29 nt crRNA (scrRNA) phosphorothioate (PS) backbone was synthesized with chemical substitutions of 2′-fluoro (2′-F), 2′-O*-*methyl (2′*-*O*-*Me) and *S-*constrained ethyl (cEt) which increased their specificity but not on-target efficiency in human cells (compared to unmodified crRNA) due to its increased binding affinity to tracrRNA and metabolic stability (Rahdar et al., 2015). These contrasting results indicate that more exploration of chemically modified sgRNAs or only crRNAs is required to reach a conclusion.

#### 3.1.5 Activity Scores of gRNA

The efficiency of guide RNA is difficult to predict due to its sequence dependency (Liu et al., 2016a), so a good option is to choose the best one from three tested gRNAs (Ran et al., 2013a) after confirmation by the T7E1 endonuclease assay (Larcher et al., 2014; Vouillot et al., 2015) and direct Sanger sequencing of the target sequence PCR products, particularly for knock-in experiments (Brinkman et al., 2014), DNA capillary electrophoresis of amplified products (Dahlem et al., 2012; Yang et al., 2015), or using fluorescent reporters (Kim et al., 2011). In many studies, guide efficiency correlating scores and sequence criteria have been identified which helps to reduce guide RNA number to test for genome editing. A list of online tools to predict the efficiency of sgRNA is shown in Supplementary Table S2. After predicting sgRNA using any of these tools, the highest score value should be obtained to increase efficiency and specificity.

### 3.2 Number of sgRNAs

The number of sgRNAs depends upon the particular application of the CRISPR/Cas system. Point mutation or knock-in can be achieved using single sgRNA along with wild-type Cas9. However, knock-in with single sgRNA and wild-type Cas9 results in many off-target effects that may destabilize genome integrity. Two sgRNAs have been used to cleave the flanking site of the target gene and replace it with a fluorescent marker to isolate the null allele in *Drosophila melanogaster* (Gratz et al., 2014) and *Caenorhabditis elegans* (Paix et al., 2015). The knock-out yield in mice can be increased to 95% by adopting a low dose triplet (three sgRNAs) rather than a high dose singlet (single sgRNA) strategy for the same target gene in oocytes (Sunagawa et al., 2016). Multiplex genome editing can be performed by simultaneously co-expressing multiple sgRNAs with Cas9. The recovery of homozygous mutants can be increased by targeting one gene with multiple sgRNAs in T0 tomato (Brooks et al., 2014) and rice plants (Xie et al., 2015; Wang et al., 2016). The doxycycline-inducible Cas9 system has been used in multiplex editing to efficiently and simultaneously delete lysine demethylase 5A, 5B, and 5C *in vitro* and *in vivo* (Cao et al., 2016).

### 3.3 Expression Level of CRISPR Components

The expression level of CRISPR components is associated with extension of culture period and individual expression level of sgRNA and Cas9. The proportion of mutated cells increases with extended culture time in soya bean somatic embryos (Jacobs et al., 2015) and rice callus infected with *A. tumefaciens* (Mikami et al., 2015). This is because of the acquisition of new mutations as well as proliferation of the existing mutants. However, the regenerative capacity of cells can be reduced by this method with an increased risk of producing chimeric plants (Xu et al., 2015b). The effect of expression level of sgRNA and Cas9 are explained below.

#### 3.3.1 Expression Level of sgRNA

It depends upon the type of promoter and the host cell line. Most studies have employed the RNA polymerase III promoter (mostly constitutive and consisting of a few cellular promoters) for sgRNA expression which makes conditional or induced expression impossible (Orioli et al., 2012). Researchers have expressed sgRNA from artificial gene RGR which produces sgRNA mRNA with a ribozyme sequence at the flanking ends after transcription following cleavage and generation of a mature sgRNA with high *in vitro* and yeast genome editing (Gao and Zhao, 2014). Multiplex genome editing in human cells has been achieved using a cell-type specific promoter and the Csy-4 dependent method to form separate mature gRNAs from the same precursor mRNA to improve genome editing efficiency (Nissim et al., 2014). A synthetic hybrid promoter consisting of tRNA and RNA polymerase III has been employed in *Yarrowia lipolytica* to enhance gRNA expression, resulting in 100% transformants by inhibiting the NHEJ process (Schwartz et al., 2016).

The sgRNA expression level is one of the determinants for increased on-target efficiency of Cas9. Higher expression levels of sgRNA and the repair template increase genome editing in rice (Sun et al., 2016), tobacco (Baltes et al., 2014), *S. cerevisiae* (Stovicek et al., 2015), *Yarrowia lipolytica* (Schwartz et al., 2016), and mammals (Ran et al., 2013a). In contrast, higher sgRNA level limited genome editing efficiency in Arabidopsis (Ma et al., 2015b) and tomato (Pan et al., 2016a). Xie et al. (2015) reported that expression levels of Cas9 and sgRNA are lower in transgenic plants or the callus compared to the protoplast, which may affect editing efficiency. Ranganathan et al. (2014) reported less off-target activity than on-target activity in a human cell line when guide RNA was expressed from a weaker H1 promoter. These results indicate species-specific editing-efficiency based on the sgRNA expression level which needs to be further confirmed using more advanced approaches.

#### 3.3.2 Expression Level of Cas9

Specificity and kinetics of gene editing can be affected by the level of Cas9 protein (Ran et al., 2013a; Pattanayak et al., 2013; Fu et al., 2014). For example, five-time drop in the level of Cas9 protein increased its specificity seven-times by affecting the on-target efficiency for just two-times (Ran et al., 2013a). Despite extensive application of CRISPR/Cas9 technology in plants for genome editing, there is variation in editing efficiencies, as the Cas9 expressing promoter, Cas9 codon optimization, and the positional effect can directly affect the Cas9 expression level and targeting efficiency (Wang et al., 2015a; Ma et al., 2015b; Yan et al., 2015; Mao et al., 2016). Constitutive Cas9 expression results in higher genome editing in mice (Platt et al., 2014), mammalian cells (Koike-Yusa et al., 2014), *S. cerevisiae* (DiCarlo et al., 2013), and rice (Mikami et al., 2015). In contrast to rice and some other higher plants, a higher Cas9 level reduces editing efficiency in mosaic Arabidopsis (Mao et al., 2013; Feng et al., 2014; Jiang et al., 2014; Ma et al., 2015b; Yan et al., 2015) and toxicity in *Chlamydomonas reinhardtii* (Shin et al., 2016) while no toxicity was observed in *Aspergillus fumigatus* with Cas9 constitutive expression (Fuller et al., 2015). Moreover, reduced search efficiency of Cas9 for heterochromatic regions indicates that access to DNA target site also contributes to specificity and efficiency (Knight et al., 2015).

Besides constitutive Cas9 expression, induced (chemical and light) and tissue-specific expression has also been evaluated in various studies to improve genome editing efficiency and specificity. In the case of CRISPR/Cas9 technology, chemically induced expression is achieved by using either an inducible promoter (e.g., doxycycline) or a Cas9 variant. Doxycycline-inducible Cas9 (iCas9) has been reported *in vivo* by genetic screening studies in humans (Wang et al., 2014); genome editing studies in mice (Dow et al., 2015); and human iPSCs (Zhu et al., 2015). This iCas9 system has also been reported *in vitro* for cell lineage specific reprogramming studies in a mouse cell line (Chakraborty et al., 2014); genomic loci imaging studies in human cell lines (Chen et al., 2013); reversible gene silencing studies in human iPSCs (Mandegar et al., 2016); multiplexed gene activations studies in cells and zygotes (Cheng et al., 2013); and biallelic gene knockout studies in hiPSCs (González et al., 2014). Cas9 variants have been developed to induce their activity in the presence of small cell-permeable 4-hydroxytamoxifen (4-HT) by combining Cas9 with either 4-HT responsive intein (Davis et al., 2015) or hormone-binding domain of the estrogen receptor (ERT2) (Liu et al., 2016b). Similarly, a light induced Cas9 system has been validated in mammalian cells to study: 1) transcriptional regulation using the p65-CRY2/dCas9-CIB1 construct (Nihongaki et al., 2015b) and the VP64-CRY2/CIB1-dCas9-CIB1 construct (Polstein and Gersbach, 2015) and 2) optogenetic control of genomic editing using the Cas9N713-pMag/nMagHigh1-Cas9C714 construct (named photoactivatable Cas9 (paCas9-1)) (Nihongaki et al., 2015a). The p65-CRY2/dCas9-CIB1 photoactivatable system constitutes sgRNAs and two fusion proteins. The first fusion protein constitutes dCas9 and CIB1; which acts like a genomic anchor probe and binds to target sequence with the help of sgRNAs. The second fusion protein constitutes transcriptional activator domain and photolyase homology region of CRY2 (CRY2PHR) which acts like an activator probe. The CIB1 and CRY2PHR are heterodimerized with the blue light irradiation and transcription is activated with the recruitment of activator domain to the target locus (Nihongaki et al., 2015b). Similarly, VP64-CRY2/CIB-dCas9-CIB1 construct constitutes light-inducible heterodimerizable proteins CRY2 and CIB1 attached to transactivation domain (VP64) and either C- or N-terminal catalytically inactive Cas9 (dCas9), respectively. This system is also directed to the target site with the help of sgRNAs and activates the transcription with blue light (Polstein and Gersbach, 2015). The Cas9N713-pMag/nMagHigh1-Cas9C714 construct constitutes photoinducible dimerization domains (named as Magnets) and split Cas9 fragments. Upon blue light irradiation, paCas9 induces genome editing by involving both homology-directed repair and nonhomologous end joining (Nihongaki et al., 2015a).

To improve spatial genome editing, tissue-specific promoters are being used in zebrafish (Ablain et al., 2015), *drosophila* (Xue et al., 2014b), mammalian cells (Yoshioka et al., 2015), and plants (Osakabe et al., 2016). Similarly, the mosaic effect in plants is reduced by using specialized promoters such as INCURVATA 2 promoter (Hyun et al., 2015), the meristem-specific YAO promoter (Yan et al., 2015), the germline-specific SPL promoter (Mao et al., 2016), the egg-cell-specific DD45 (Mao et al., 2016), and EC1.2 promoters (Wang et al., 2015b). For effective Cas9 expression in human iPSCs, EF1α promoter is proved stronger than CAG promoter (Matsui et al., 2014), while CMV and SV40 are not recommended due to transcriptional silencing (Hotta and Ellis, 2008). Programmable and multiplexed regulation of various gene networks in human cells is achieved by combining the RNA regulatory strategies with Cas9 based transcription factors (Nissim et al., 2014). For example, immune responses against transgene products can be minimized by using the mir-142-3p which can repress the expression of associated cellular transcripts by binding to their target sequences (Majowicz et al., 2013).

### 3.4 Delivery Methods

#### 3.4.1 For Cas9 DNA

Cas9 is a sequence-specific endonuclease that is delivered to the host cell or organism as DNA, mRNA, or protein for a genome editing experiment. The first human genome editing was performed in 2013 in which all CRISPR components were delivered in the form of DNA plasmids and/or expression cassettes (Mali et al., 2013b). With advancements in the field, various vectors and methods have been attempted to achieve maximum editing efficiency and precision. For example, five-fold more HDR efficiency was achieved with a particle bombardment technique in rice (Sun et al., 2016) and maize (Svitashev et al., 2015) compared to Agrobacterium-mediated transformation. Enhanced genome editing was observed by 1) introducing components using adenovirus vector (AV) or adeno-associated vector (AAV) in mice (Senís et al., 2014; Swiech et al., 2015; Rodriguez et al., 2016) and a lentivirus into melanoma cell lines (Shalem et al., 2014) and 2) using the hydrodynamic injection technique in mice (Xue et al., 2014a). Mostly transfection methods such as electroporation or lipofectamine are used to transfer CRISPR components into host cell (Costa et al., 2007) but nucleofector is commonly used for human iPS cells genome editing (Ran et al., 2013b).

AAV vectors are common delivery tools due to safety and efficiency (Kaufmann et al., 2013; Kotterman and Schaffer, 2014; Kotterman et al., 2015). One problem is the size of the commonly used SpCas9 gene, which cannot be accommodated in wild type AAV, so smaller orthologues (Esvelt et al., 2013) or engineered AAV (Kotterman and Schaffer, 2014) are used as a solution. Moreover, orthologues have been developed to simultaneously perform multiplexed RNA-guided transcriptional repression, activation, and gene-editing (Esvelt et al., 2013). Similarly, AAV vector has shown some efficacy in multiple monogenic disorders including choroideremia (MacLaren et al., 2014), haemophilia B (Nathwani et al., 2011), and Leber’s congenital amaurosis type 2 (Hauswirth et al., 2008; Bennett et al., 2012). Lentiviral vectors have been used to study 1) drug resistant and cell viability genes via loss-of-function mutation in mouse and human cells (Koike-Yusa et al., 2014; Shalem et al., 2014; Wang et al., 2014; Zhou et al., 2014); 2) leukemia causing genes in mouse hematopoietic cells (Heckl et al., 2014); 3) the role of p53 and Pten in the formation of liver tumors using mouse 3T3 cells (Xue et al., 2014a) and 4) Kras gain-of-function mutation (KRAS G12D) in the Neuro-2a neuroblastoma cell line (Platt et al., 2014).

#### 3.4.2 For Cas9 mRNA and Protein

Cas9 and sgRNA show persistent expression when delivered in the form of a cloned vector (Xue et al., 2014a; Yin et al., 2014; Yang et al., 2016); however, it might cause mutation with random integration of CRISPR components at on-target and off-target sites into the host genome (Cradick et al., 2013; Fu et al., 2013). The Cas9mRNA/sgRNA or Cas9protein/sgRNA is injected into the host embryo or zygote for specific genome editing. For example, the injection of sgRNA and Cas9mRNA causes 1) targeted mutagenesis in 88% of embryonically injected flies with a 33% transmittance rate (Bassett et al., 2013), 2) generation of one-step multiple allelic mutated mice *via* zygotic injection (Wang et al., 2013), 3) correction of mouse crygc allele (Wu et al., 2013) and dystrophin gene (Long et al., 2014) via zygotic injection, and 4) correction of human beta-hemoglobin gene via tripronuclear zygotic injection (Liang et al., 2015). Similarly, genome editing efficiency and specificity can be increased by 1) using the Cas9 protein and sgRNA delivered by electroporation (Kim et al., 2014), protein transduction (Ramakrishna et al., 2014), and lipofection (Zuris et al., 2015), 2) using the MS-modified sgRNA and Cas9 protein complex (Hendel et al., 2015), 3) synchronizing the delivery of ribonucleoprotein (RNP) with the stage of cell cycle in human cells (Lin et al., 2014a) and 4) using a solubilizing salt solution for the fluorescent Cas9/sgRNA complex in zebrafish embryos (Burger et al., 2016). The mixture of Cas9 mRNA and gRNA (Zhang et al., 2016b) or pre-assembled mixture of RNP (Woo et al., 2015; Svitashev et al., 2016; Liang et al., 2017) can be delivered to plant cells for efficient genome editing. These delivery methods are summarized in Supplementary Table S3.

### 3.5 Features of Target Sequence

#### 3.5.1 GC Content

GC content is related to genome size in bacteria, but it is very complex in eukaryotes due to repetitive DNA segments and isochores. There is huge variation in GC content among microbes but less of a range in plants and animals, e.g., monocots and vertebrates have higher GC content than dicots and invertebrates, respectively. There is more GC content in animal chromosomes than that in plants but no correlation between chromosome size and GC content has been observed (Li and Du, 2014). In the case of CRISPR/Cas technology, target sequence GC content affects genome editing efficiency (Zhang et al., 2014; Ma et al., 2015b). For example, higher GC content (>70%) facilitates hybridization between the spacer and the protospacer, which may increase off-targets (Lin et al., 2014b; Tsai et al., 2015; Li et al., 2016). GC content lower than 30% also results in higher off-targets (Fu et al., 2013; Pattanayak et al., 2013). However, no off-targeting was observed with GC content of 57% in soybeans (Jacobs et al., 2015); 50% in Arabidopsis (Sauer et al., 2016); and 50–70% in rice (Ma et al., 2015b).

#### 3.5.2 Stem Loop Structure

The genomic sites of the host target sequence that pair six continuous nucleotides with the sgRNA scaffold should not be selected because it results in the formation of a stem loop structure between the sgRNA scaffold and target sequence, which hinders perfect binding with the target sequence ultimately leading to less efficient genome editing (Ma et al., 2015b).

#### 3.5.3 Chromatin State and Strand Preference

DNA exists in two chromatin states, i.e., euchromatin (less condensed) and heterochromatin (highly condensed); however, accessibility to the latter is a bit difficult. Thus, Cas9 targeting a DNA site in the highly condensed portion leads to decreased binding (Wu et al., 2014; Verkuijl and Rots, 2019), which may be due to less accessibility to the PAM sequence (Hinz et al., 2015). The heterochromatin state of the target sequence can reduce the diffusion of Cas9 (Knight et al., 2015) and CRISPR-Cas9 mediated mutagenesis by 7-fold (Kallimasioti-Pazi et al., 2018). The presence of epigenetic modifications or transcription factors may also affect Cas9 binding following genome editing. It has also been observed that sgRNAs targeting non-transcribed DNA strands are more effective compared to those targeting transcribed DNA strands (Wang et al., 2014). The cleavage efficiency of SpCas9 is independent of methylation of the target region (Ran et al., 2013a).

## 4 Factors Contributing to Donor-Based CRISPR Editing

### 4.1 Host DNA-Repair Pathways

The genome is continuously facing damages by various factors, ranging from metabolic byproducts to UV radiation. These factors affect the target genome by their own ways. One of them is the breakage of DNA phosphodiester backbone in either single strand or both strands (DSBs). These DSBs are mainly repaired by the NHEJ rather than HDR DNA-repair pathway (Figure 3). Integration of foreign DNA into the target genome by CRISPR/Cas technology is based on the principle of homologous recombination which is least prominent in the presence of NHEJ (as explained earlier); thus, either inhibiting NHEJ or stimulating HDR may lead to improved knock-in efficiency. To improve donor-based editing, various researchers have tried to modify the priorities of these naturally competing pathways. For example, the NHEJ pathway can be inhibited by inhibiting key components of the NHEJ pathway, such as DNA ligase IV, KU80, or KU70 in plants and animals (Chu et al., 2015; Maruyama et al., 2015; Endo et al., 2016). Stimulating the HDR pathway using RS-1 (HDR enhancer) also led to a two-to five-fold improved gene knock-in at various loci (Song et al., 2016). Yu et al. (2015) revealed that certain small chemical molecules can modulate the NHEJ and HDR pathways to improve genome editing. Moreover, NHEJ occurs throughout the cell cycle (Panier and Boulton, 2014) whereas HDR happens only during the G2 and S phases (Mali et al., 2013b; Wang et al., 2013; Yang et al., 2013), so transgenic Cas9 expression in meiotic cells led to high knock in efficiency due to higher HDR than NHEJ during gene drive experiments conducted in mosquitos (Gantz et al., 2015).

**FIGURE 5:**
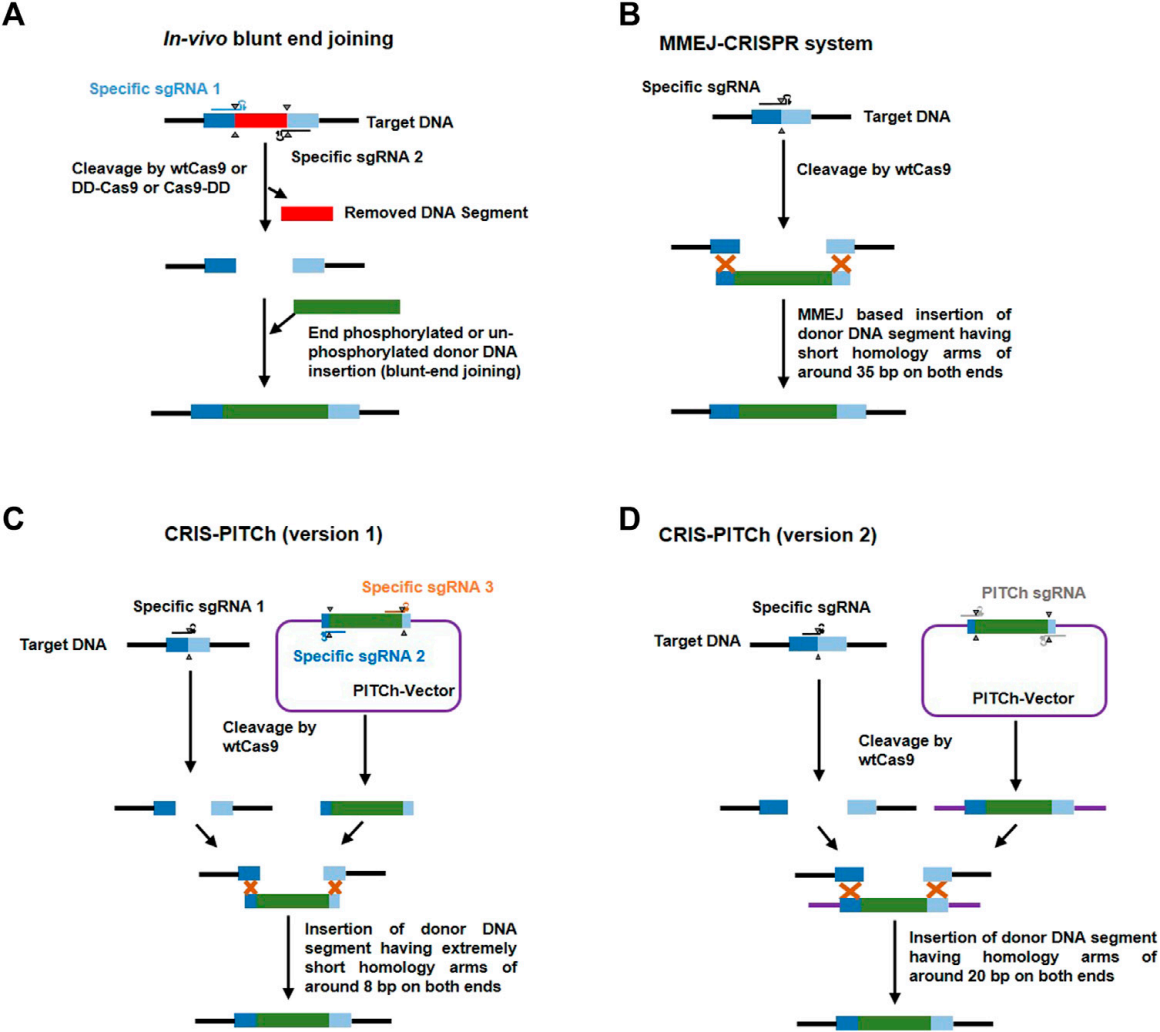
Representative methods in donor-based host genome editing. **(A)** In vivo blunt-end joining involves using two target specific sgRNAs (blue and black) to cleave target DNA [releasing a DNA segment (red)] with either wildtype cas9 (wtCas9) or Cas9 attached to the protein destabilization domain (DD) like FKBP12-L106P which destabilizes Cas9 in the absence of a stabilizing agent, such as Shield-1 (Clontech); it leads to precise knock-in of donor DNA (green) *via* non-homologous end joining (NHEJ). **(B)** The MMEJ-CRISPR system involves cleavage of target DNA using one specific sgRNA (black) and wtCas9; and integration of a donor DNA fragment (with around 35 bp homologous arms at both ends (dark and light blue) at the cut site through the process of microhomology-mediated end joining (MMEJ) during the M-early S phases of the cell cycle. **(C)** CRIS-PITCh version 1 involves three specific sgRNAs [one to cleave target DNA (black) and the other two (yellow and brown) to cleave the DNA sequence flanking outside the homologous sequence (8 bp) in the donor vector (PITCh-vector) which releases a donor DNA segment with homologous arms (dark and light blue)] and wtCas9 to precisely integrate any gene of interest without end trimming by proximal MMEJ. **(D)** CRIS-PITCh version 2 also involves three sgRNAs [one specific sgRNA (black) for host target genome cleavage and two general PITCh sgRNAs (grey) to cleave the DNA sequence flanking outside the homologous sequence (20 bp) in the donor vector (PITCh-vector) which releases a donor DNA segment with homologous arms (dark blue and light blue)] and wtCas9 to precisely integrate any gene of interest with end trimming by distal MMEJ.

About 90% of DSBs generated in rapidly growing mammalian cells by ionizing radiation and Cas9 are repaired within 1 h (Metzger and Iliakis, 1991) and 15 h (Kim et al., 2014), respectively. This finding suggests a long lifetime (approximately 6 hour) of the Cas9-DNA complex. A close analysis of this complex revealed asymmetric cleavage and release of the 3′end of the non-targeted DNA strand by sgRNA. Richardson et al. (2016) achieved an improved knock-in efficiency in human cells using single stranded DNA (ssDNA) donors complementary to the first released strand. The donor-based knock-in can also be executed by the microhomology-mediated end joining (MMEJ) pathway (Figure 5) which is explained below.

### 4.2 Specifications of Donor DNA

In CRISPR research so far, donor DNA has been used either in the form of a plasmid or single-stranded oligodeoxyribonucleotides (ssODNs), each having its own efficiency in particular target species. For example, using a donor in the form of ssODNs instead of a plasmid resulted in HDR efficiency from 10 to 80% in mice (Yang et al., 2014). In plants, ssODN donor showed ≈5% gene conversion (unidirectional interchange between similar sequences) efficiency in Arabidopsis (Sauer et al., 2016); a plasmid donor showed 9% gene replacement efficiency in *Nicotiana benthamiana* (Li et al., 2013); a ssODN donor (ssOligo2; 127 nt) showed 0.4% (4/1,000) mutation frequency in maize compared to 0.2% (2/1,000) using a plasmid donor (794 bp) (Svitashev et al., 2015); insertion frequency of ≈1 kb homologous arms containing the expression cassette was 4% in maize (Svitashev et al., 2015) and 4.6% in soybean (Li et al., 2015b); and the gene-replacement frequency in Arabidopsis was 0.8% using 733 and 825 bp homologous arms containing a 3.9 kb donor plasmid (Zhao et al., 2016). Moreover, increased editing efficiency and longer (100 bp) donor insertions have been achieved at ROSA loci of rats and mice using phosphorothioate-modified ssODNs (Renaud et al., 2016).

Normally, 700–1,000 bp length of a homologous sequence should be added to both sides of the donor cassette and the added sequence must be adjacent to but not include the sgRNA target site (Yusa, 2013). Modified systems have been developed for gene knock-in at the target region using donors with small homologous sequences or those without any such sequences. For example, Zhang et al. (2016a) introduced MMEJ of the donor construct into the host target genome and named it the MMEJ-CRISPR system. This system is active only during the M and early S phases when HR is inactive (Taleei and Nikjoo, 2013). They added approximately 35 bp of a homologous sequence to donor and achieved 95–100% accuracy in *A. fumigatus* and also demonstrated that this system is not dependent on the ku80 pathway (Figure 3). Sakuma et al. (2016) exploited the MMEJ phenomenon to introduce a more user-friendly donor based genome editing system known as CRIS-PITCH [two versions, i.e., CRIS-PITCh (v1) and CRIS-PITCh (v2)]. These PITCh vectors require a few days to construct and can successfully complete the knock-in process within 1 week in frog embryos and within 1.5 months in human cell lines. Geisinger et al. (2016) achieved highly precise genome editing in mouse and human cells *via in vivo* blunt-end cloning with the use of wt-Cas9 and Cas9 attached to the protein destabilization domain (FKBP12-L106P). Schematic illustrations of the MMEJ-CRISPR system, the CRIS-PITCH system and *in vivo* blunt end cloning are shown in Figure 5.

### 4.3 Cas9 Versions for Specificity Improvement

The target-specific sgRNAs may show some off-target effects that disturb the overall stability of the genome and knock-in of the donor cassette. This occurs because of the two endonuclease domains of the wild-type Cas9 enzyme; each cuts the opposite strand of the DNA resulting in DSBs. To minimize these hazardous effects, one of the domains is inactivated by a mutation (D10A or H840A) to form nickase Cas9 (nCas9). A nick is generated on opposite strands of two closely located target sites using pairs of sgRNAs and nCas9, which minimizes the off-targets by maintaining on-target cleavage efficiency (Cong et al., 2013a; Mali et al., 2013a). The paired Cas9 nickases recognize and cleave four 100 bp apart target sites on opposite strands (Ran et al., 2013a; Shen et al., 2014). Although some studies have reported fewer indel mutations using nCas9 with single sgRNA, paired sgRNAs with nCas9 have been observed with reduced off-targeting in human cells and mice (Sander and Joung, 2014). The gRNAs in the PAM-out orientation have more efficiency than those in the PAM-in orientation. Similarly, D10A nCas9 is more efficient than H840A nCas9 (Ran et al., 2013a).

The off-targets of monomeric nucleases can be minimized by using the dimerization-dependent RNA-guided FokI-dCas9 nucleases (RFNs) in which dCas9 is fused with FokI nuclease to form fCas9. This fCas9 uses a pair of sgRNAs targeting opposite strands of DNA with targets separated by 15–25 bp in a “PAM-out” orientation. This system shows a dramatic increase in specificity with comparable efficiency to that of nickase Cas9, which is two-thirds that of wild-type Cas9 (Guilinger et al., 2014; Tsai et al., 2014). Genome editing specificity can also be increased by combining two orthogonal approaches (independent approaches adopted to conclude via getting same or different results). For example, using tru-RFNs, made by combining truncated gRNAs (trugRNAs) with RFNs, results in increased specificity and efficiency in embryonic stem cells and human cancer cell lines (Wyvekens et al., 2015). Despite increased specificity, these methods are relatively complex and require more combination of guide RNAs that may have to be evaluated.

The precision in target recognition can also be enhanced by the fusion of programmable DNA-binding domain (pDBD) of other nucleases such as TALENs or ZFNs. For example, SpCas9^MT3^-ZFP^TS2:TS3:TS4^ was constructed by linking the mutated (R1335K) Cas9 (SpCas9^MT3^) with programmed zinc-finger protein (ZFP^TS2:TS3:TS4^), to target the closely spaced genomic DNA sites. The SpCas9^MT3^ version of Cas9 prefers NGN PAM while ZFP^TS2:TS3:TS4^ constitutes three different ZFPs to recognize sequence surrounding the guide RNA target sites 2, 3, and 4. This engineered Cas9 provides 150-fold increased specificity because of cooperative association between the two separate DNA binding events like the above-mentioned nickases and FokI (Bolukbasi et al., 2015). However, these strategies are less applicable because they require larger transgenes and additional components.

Recently, rational engineering of SpCas9 and SaCas9 has been done based on their crystal structures; the engineered versions showed enhanced specificity and thus named as eSpCas9 and eSaCas9, respectively. In eSpCas9 and eSaCas9, three and four positively charged residues of non-targeted DNA strand groove were neutralized by replacing them with alanine, respectively (Slaymaker et al., 2016). These mutations allow competitive rehybridization of DNA with the invasion of gRNA on target strand by weakening the protein binding on non-target strand. This increase in stringency between RNA and DNA matching dramatically reduced the genome-wide off-targeting. Surprisingly, on-target efficiency of eSpCas9 was comparable to that of wild-type SpCas9. A similar strategy was followed by another research group to design “high-fidelity SpCas9” (SpCas9-HF1) in which residues interacting with phosphate backbone of target DNA strand have been substituted with four alanine residues, unlike eSpCas9 (Kleinstiver et al., 2016). The on-target efficiency of SpCas9-HF slightly varies for tested guides as compared to that of eSpCas9; but, it is comparable (>70%) to that of wild-type (86%). Similarly, high-fidelity (HiFi) Cas9 variant was identified via unbiased bacterial screening approach in which a single point mutation (R691A) caused reduced off-targeting while maintaining higher on-target activity as a RNP complex (Vakulskas et al., 2018). Along with these, few more high-fidelity SpCas9 variants have been developed such as FeCas9 (Yin et al., 2019), evoCas9 (Casini et al., 2018), HypaCas9 (Chen et al., 2017), SpCas9^2Pro^ (Babu et al., 2019) LZ3 (Schmid-Burgk et al., 2020), and Sniper-Cas9 (Lee et al., 2018).

Another approach to engineer Cas9 is alteration in its PAM recognition sequence. This might increase the number of genome-wide targets as well as improve specificity by requiring less-abundant PAM or PAM with longer sequence across the genome. One strategy is to replace PAM-interacting domain (PID) of recipient with that of ortholog which recognizes a different PAM sequence. This has been done for *Streptococcus thermophiles* CRISPR-3 Cas9 (St3Cas9) and SpCas9 which retained their genome editing function (Fonfara et al., 2013; Nishimasu et al., 2014). Continuous phage-assisted evolution of SpCas9 generated a SpCas9 variant (xCas9) which can recognize broader range of PAM sequences including NG, GAT, GAA (Hu et al., 2018). Directed evolution has also been used to change the PAM specificity of SaCas9 (Kleinstiver et al., 2015a) and SpCas9 (Kleinstiver et al., 2015b). Remarkably, only four mutations brought about engineered “VRER SpCas9” with specificity for the PAM sequence “NCGC” which has 23 times less abundance in human genome than NGG. Directed evolution caused a point mutation (D1135E) in SpCas9 which increased its specificity for NGG over NAG PAM (Jiang et al., 2013a; Hsu et al., 2013; Kleinstiver et al., 2015b). In case of SaCas9, targeting range is increased by 2–4 fold after modifying its PAM from NNGRRT to NNNRRT via directed evolution (Kleinstiver et al., 2015a). Few more PAM-flexible variants have also been reported including SpCas9-NG (Nishimasu et al., 2018), SpG and SpRY (Walton et al., 2020). Overall, all these engineered versions of Cas9 improve their specificity to target without sacrificing their efficiency much.

## 5 Factors Contributing in Epigenome Editing, Genome Imaging, and Protein-Genome Interaction

### 5.1 Dead Guide

A type of guide sequence causing efficient binding of wild-type Cas9 to its target sequence without inducing cleavage is known as “dead guide (dRNA).” Researchers successfully synthesized these dRNAs by shortening the length of the normal guide to 14 or 15 nt and achieved good transcriptional control when co-transfected with wild-type Cas9 fused with a transcription-activating domain (Dahlman et al., 2015; Kiani et al., 2015). Moreover, orthogonal gene activation and knockout can be achieved by co-expressing wild-type Cas9 with dead guide (14 or 15 nt) and normal guide (20 nt), respectively, in the same cell (Dahlman et al., 2015). Although decreasing the length of guide can increase off-targets, the chances of transcriptional modulation at these sites may be low due to the presence of few off-targets within the transcriptional start site.

### 5.2 Dead or Deactivated Cas9 (dCas9)

The expression level of genes is naturally controlled by two main epigenetic marks, acetylation and methylation, executed by associated proteins (Javaid and Choi, 2017). dCas9 is generated by point mutation-mediated inactivation of both Cas9 catalytic domains, which does not affect its RNA-guided DNA binding ability (Jinek et al., 2012a). It can be used for epigenome editing (CRISPRa and CRISPRi systems) and genome-imaging. Moreover, various transcription factors (for example those involved in reprogramming) interact with target DNA in a specific manner (Yesudhas et al., 2017b). This interaction can be further validated by using the dCas9 version of the CRISPR endonucleases.

#### 5.2.1 CRISPR Activation

CRISPRa is achieved by fusing the dCas9 enzyme to transcriptional activators, such as an ω subunit of RNA polymerase in bacterial cells (Bikard et al., 2013) and p65AD or VP64 in mammalian cells (Mali et al., 2013a; Gilbert et al., 2013; Konermann et al., 2013; Perez-Pinera et al., 2013). Various strategies have been adopted to enhance the efficiency of a CRISPRa system by recruiting multiple transcriptional activators: 1) In addition to multiple sgRNAs at a single promotor to recruit multiple activators (Cheng et al., 2013; Maeder et al., 2013; Chavez et al., 2015), strategies to recruit multiple activators to the dCas9 binding site have been developed (Tanenbaum et al., 2014; Chavez et al., 2015; Konermann et al., 2015). For example, the synergistic activation mediator system allows multiple activators to work synergistically by using both sgRNA and dCas9 as scaffolds (Konermann et al., 2015). In this system, sgRNA modified with two MS2 RNA aptamers is combined with dCas9-VP64. Each aptamer recruits a pair of similar RNA binding proteins, MCPs (MS2 bacteriophage coat proteins), which are bound with p65-and HSF1-activating domains (MCP-p65-HSF1) (Konermann et al., 2015; Nishimasu et al., 2015). This system has been applied for large-scale genome screening because of its increased efficiency (Konermann et al., 2015). 2) Multiple VP64 activators can be recruited to a single dCas9 binding site by combining the dCas9 system with multipeptide array, such as SunTag. For example, fusion of dCas9 to the polypeptide array (GCN4s) recruits multiple (10 or 24) copies of its cognate scFv (single-chain variable fragment), an engineered portion of the anti-GCN4 antibody. This scFv was further fused to VP64 which eventually led to strong upregulation of the target gene [chemokine (C-X-C motif) receptor 4 (CXCR4)] because of multiple VP64 copies per dCas9 (Tanenbaum et al., 2014). This system has also been used to reduce cell growth by upregulating the expression of CDKN1B (cyclin-dependent kinase inhibitor 1B) (Tanenbaum et al., 2014) and gain-of-function screening at the genome scale (Gilbert et al., 2014). 3) A tripartite activator system has been developed by fusing three various activators in tandem to dCas9 [VP64-p65-Rta (VPR)] which showed greater activation efficiency than dCas9-VP64 when used with multiple sgRNAs (Chavez et al., 2015). Reprogramming of target cells can be achieved by using multiple approaches (Anwar et al., 2016; Lee et al., 2017) and CRISPR activation system is one of them (Balboa et al., 2015). All of these systems mimic the intrinsic mechanism of gene activation by recruiting multiple activators at the target site (He and Weintraub, 1998; Govind et al., 2005).

Similarly, dCas9 can be used to recruit epigenetic modifiers at a given locus to reshape the epigenome. For example, *Neisseria meningitidis* dcas9 (Nm dCas9) fused to histone demethylase LSD1 can reduce the expression of genes (which are controlled by the targeted enhancers) by decreasing the epigenetic marks H3K27ac and H3K4me2 near the targeted enhancer region (like Oct4 and Tbx3) (Kearns et al., 2015). Fusing the catalytic core domain of histone acetyltransferase p300 with dCas9 (Nm dCas9-p300 core and Sp dCas9-p300 core) can be used to activate the transcription of various endogenous genes by increasing the H3K27ac level at the targeted enhancer or promoter regions (Hilton et al., 2015).

#### 5.2.2 CRISPR Interference

The dCas9 mediated regulation of gene expression is executed by recruiting various proteins and RNA factors at the target site (Gilbert et al., 2013; Qi et al., 2013). Transcriptional inhibition by the CRISPR machinery is termed CRISPRi and only dcas9 is sufficient in bacterial cells via steric hindrance of the transcriptional machinery (Bikard et al., 2013; Qi et al., 2013). However, it is not very effective in mammalian cells unless dCas9 is fused to a transcriptional repressor domain (such as KRAB of Kox1) (Gilbert et al., 2013; Konermann et al., 2013).

#### 5.2.3 Genome Imaging

One of the important things in genome biology is to understand the correlation between linear genetic information imprinted on DNA and its three-dimensionally compact organization inside the cell nucleus because many studies have highlighted the impact of organization on the regulation of gene expression and cell differentiation (Lanctôt et al., 2007; Schneider and Grosschedl, 2007; Peric-Hupkes et al., 2010; Dixon et al., 2015). Because of the lack of a proper tool, it was difficult to visualize genomic dynamics in a sequence-specific manner. However, it has become possible with the sequence-specific binding of the dCas9 regardless of the genome architecture and epigenetic state. For example, fusing *Streptococcus pyogenes* dCas9 (Sp dCas9) to enhanced GFP can be used to visualize the repetitive and nonrepetitive genomic loci in living human cells (Chen et al., 2013). A similar approach was used to label the endogenous telomeres, pericentric regions, and centromeres (Anton et al., 2014). dCas9-based genome imaging has been expanded with various improvements. The SunTag peptide array with dCas9 was exploited to successfully amplify imaging strength (Tanenbaum et al., 2014). Multicolor genome imaging was achieved by individually tagging St1 dcas9, Nm dCas9, and Sp dCas9 with differently colored fluorescent proteins (FPs) and targeting each of them to different loci by the corresponding sgRNAs (Ma et al., 2015a). Fixed cells and tissues can be labeled by *in vitro*-assembled complexes of fluorescently labeled dCas9-sgRNA, a technique known as CASFISH (Deng et al., 2015). Using a single particle tracking method with fused dCas9 and the HaloTag system, it was found that there is a three-dimensional diffusion based analysis of genome by dCas9, with transient binding at off-targets and reduced efficiency to search at heterochromatic regions (Knight et al., 2015).

#### 5.2.4 Protein-Genome Interaction

The sole or complex of different transcription factors interact with the target DNA by recognizing some consensus sequences (Yesudhas et al., 2017a; Yesudhas et al., 2019). Endogenous proteins interacting with a specific genome region can be found by using the CRISPR-based engineered DNA-binding molecule-mediated ChIP (enChIP) method. The loci specific proteins are pulled down by targeting the affinity-tagged dCas9 at that region using associated sgRNAs. This system has been successfully tested to analyze proteins interacting with an interferon-γ-responsive promoter (Fujita and Fujii, 2013; Fujita and Fujii, 2014).

### 5.3 Scaffold RNA System

The scRNA system is made by modifying sgRNA, instead of dCas9, to turn it into a scaffold to recruit various transcriptional regulators (Mali et al., 2013a; Konermann et al., 2015; Shechner et al., 2015; Zalatan et al., 2015). The orthogonal RNA aptamers are fused to the sgRNAs which recruit various RNA-binding proteins (RBPs) further fused to transcriptional repressors or activators. The multimodal gene regulation (i.e., simultaneous repression or activation) within the same cell can be achieved by coupling RNA aptamer-RBP pairs (such as PP7-PCP, MS2-MCP, and com-Com) to different sgRNAs and directing them to the target site with the help of dCas9 (Zalatan et al., 2015).

## 6 Applications of CRISPR/Cas System

**FIGURE 6:**
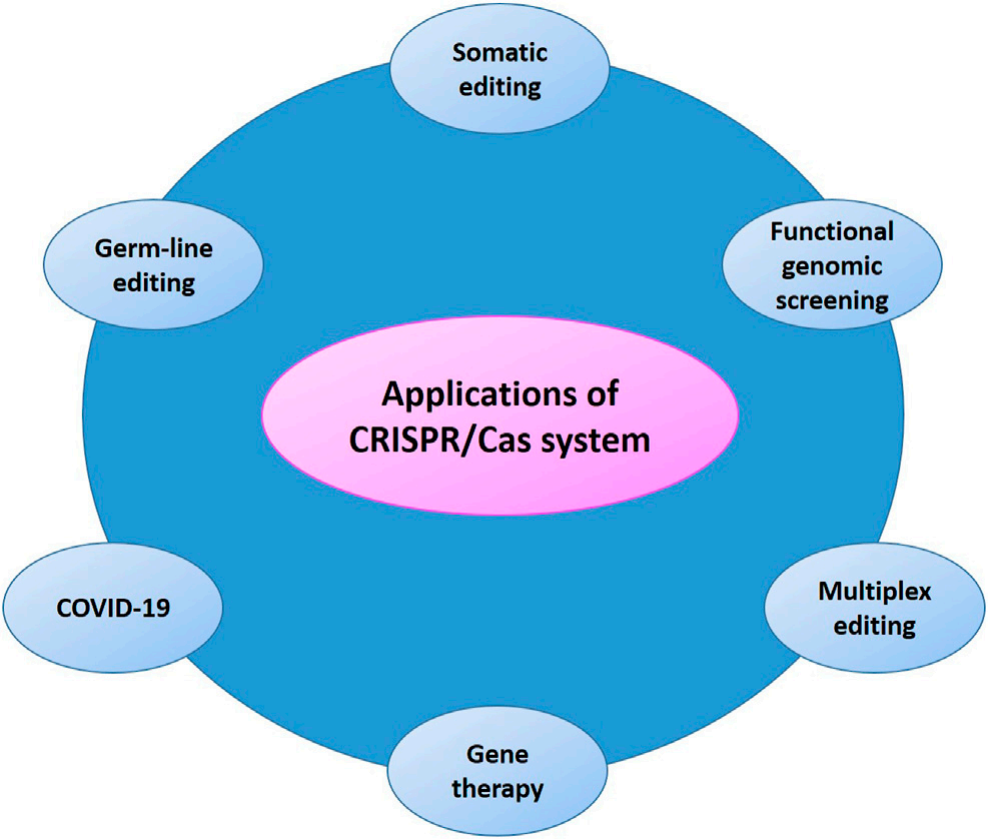
Applications of CRISPR/Cas system.

Due to its flexibility, convenience and precision, CRISPR/Cas system is preferred over previously developed genome-editing tools (such as TALENs and ZFNs) for various applications (Figure 6). Gene targeting in embryonic stem cells by homologous recombination is used to modify animal genome which played a significant role in reverse genetics with reference to diseases. It has limited application due to lack of embryonic stem cells and long-time. Recently, precise germline-modifications of various model organisms have been achieved by CRISPR/Cas technology which has revolutionized the therapeutic industry (Hwang et al., 2013; Wang et al., 2013; Niu et al., 2014; Platt et al., 2014; Whitworth et al., 2014). Rapid, simple and scalable *In vivo* modification of target genes is achieved by microinjection of customizable sgRNA and Cas9-encoding mRNA into zebrafish embryos at one cell-stage (Chang et al., 2013; Hwang et al., 2013). The highly efficient biallelic mutations in mice is done by coinjecting sgRNAs targeting multiple genes and Cas9 mRNA into mouse zygotes (Wang et al., 2013). Targeting two sites of the same gene by respective sgRNA is used to generate mice with deleted mutations (Wang et al., 2013). Moreover, genetically modified knockin mutant mice has been generated by Cre-dependent Cas9 with simple injection of sgRNA (Platt et al., 2014). Along with mice, CRISPR/Cas technology has been used to genetically engineer other model organisms which include *Caenrhabditis elegans* (Friedland et al., 2013), Drosophila (Bassett et al., 2013; Gratz et al., 2013), Axolotl (Flowers et al., 2014), rat (Hu et al., 2013), *Xenopus tropicalis* (Blitz et al., 2013; Nakayama et al., 2013), and pig (Whitworth et al., 2014). Notably, this technology has also been used to modify the genome of cynomolgus monkey (Niu et al., 2014). The development of diverse model organism with various genomic modifications will help to develop therapeutic strategies for multiple human diseases.

CRIPSR/Cas technology can also be used for somatic genome editing in various mouse models which is a tool for fast functional analysis of genes responsible for a particular disease (Xue et al., 2014a; Maddalo et al., 2014; Sánchez-Rivera et al., 2014). Hydrodynamic-based delivery of Cas9 and Pten targeting sgRNAs, with or without p53 mutation, in liver revealed the effects of p53 and PTEN knockouts (Xue et al., 2014a). The mouse model with Eml4-Alk-mediated lung cancer has been developed after Eml4-Alk inversion (chromosomal rearrangement) with CRISPR/Cas9 system (Maddalo et al., 2014). These tumors have typical molecular and histopathological characteristics of human ALK (+) non-small cell lung cancer with a sensitivity to ALK targeting inhibitors (Maddalo et al., 2014). This highlights the reliability of this technology to mutate oncogenes and tumor suppressor genes in somatic cells, which provides new approach for the development of respective disease models.

Functional genomic screening is a process to identify the function of a gene in a particular cellular process. Previously used RNA interference (RNAi) technique has limited applications because of multiple off-targets (Jackson et al., 2003; Chang et al., 2006; Adamson et al., 2012; Sigoillot et al., 2012). Moreover, RNAi cannot silence nuclear RNAs. On the other hand, CRISPR/Cas technology has a lot of success in loss-of-function screening at genomic scale (Findlay et al., 2014; Shalem et al., 2014; Wang et al., 2014). Most of the expected genes in DNA mismatch repair pathway have been identified by using lentiviral sgRNA library at genomic-scale (Wang et al., 2014). Genes responsible for resistance to late-stage melanoma drug, vemurafenib, and cell viability in pluripotent stem cells and cancer has been screened by using GeCKO (genome-scale CRISPR-Cas9 knockout) library (Shalem et al., 2014). Genes involved in cellular response to diphtheria and anthrax toxins have been screened by using CRISPR/Cas-based knockout library (Zhou et al., 2014). Functional screening of both *trans*-acting factors and *cis*-regulatory elements at high resolution in the genome can be done by linking CRISPR/Cas technology with multiplex HDR by using complex library of donor templates (Findlay et al., 2014). Two other powerful tools for functional screening are CRISPR-mediated activation (CRISPRa) and CRISPR-mediated repression (CRISPRi). Their libraries have been employed to map complex pathways by screening essential genes involved in tumor suppression, growth, differentiation, and sensitivity to various toxins (Mali et al., 2013a; Gilbert et al., 2013; Maeder et al., 2013; Perez-Pinera et al., 2013; Gilbert et al., 2014). The results promise the utilization of this tool to identify essential endogenous genes for various biological processes.

The precise and easy multiplexing ability of CRISPR/Cas system in human cells than other genome editing tools (such as ZFNs and TALENs) has made it popular. This feature is exploited for simultaneous editing of PVALB and EMX1 loci in human cells; this is done by expressing both targeting sgRNAs in a single expression cassette (Cong et al., 2013b). The simultaneous expression of two sgRNAs resulted in the deletion of 19-bp segment (Mali et al., 2013b). For multiplex engineering, vector systems for the expression of multiple sgRNAs simultaneously have been developed. For example, plasmid carrying expression cassettes of two to seven gRNAs showed editing efficiency from 4.3 to 37.8% (Sakuma et al., 2014). In another study, four gRNAs under different promoters are assembled in a single lentiviral vector *via* Golden Gate assembly. This construct is used to simultaneously edit one locus in each AAVS1 and HBG1 and two loci in IL1RN with editing efficiency of 4.8–18.4% in fibroblasts and 17.9–33.3% in HEK293T cells (Kabadi et al., 2014).

Genetic therapies are considered as powerful tool to cure monogenic diseases, cancers, HIV, or degenerative diseases. One of the exciting applications of CRISPR/Cas technology is its ability to cure genetic diseases. The dominant mutation of Crygc gene in cataracts mouse model has been corrected by simultaneous injection of mutant Crygc targeting sgRNA and Cas9 mRNA into zygotes (Wu et al., 2013). Subsequent study revealed 100% efficient production of offsprings with corrected phenotype by correcting Crygc gene in spermatogonial stem cells with CRISPR/Cas system (Wu et al., 2015). Development of muscular dystrophy in mutant mice has been prevented by correcting the dystrophin gene in germ line with coinjection of sgRNA, Cas9 and donor template into mouse zygotes (Schwank et al., 2013). The mutated locus of cystic fibrosis transmembrane conductor receptor (CFTR) in cultured intestinal stem cells derived from human cystic fibrosis patient has been correcting after homologous recombination with the help of CRISPR/Cas technology (Long et al., 2014).

Considering antiviral adaptive role of CRISPR/Cas system in bacteria, it can be used to eliminate genomes of pathogens from patients in order to cure them from various infectious disease. The new HIV infection is prevented by eliminating the HIV-1 genome from patients (Ebina et al., 2013; Hu et al., 2014). Upon transfecting the HIV-1 long terminal repeats (LTR)-specific sgRNA into human cells integrated with HIV-1 provirus, LTR target sites are cleaved and mutated which suppressed the expression of various viral genes. Additionally, it also eliminated the viral genes from chromosome of host cell (Ebina et al., 2013). Recently, the precise editing of HIV-1 genome with Cas9/sgRNAs has also been done which immunize the cells for the prevention of further infection (Hu et al., 2014).

In 2018, first Phase 1 CRISPR clinical trial opened in the United States for cancer immunotherapy to edit autologous T cells against multiple tumors. During this, T lymphocytes collected from patients’ blood were engineered *ex vivo* to knockout *α* and *β* chains of endogenous T cell receptor (TCR). Later, NY-ESO-1 antigen specific TCR encoding gene was delivered to these cells via lentiviral transduction system (Baylis and McLeod, 2017). The first clinical trial in the United States to mediate gene disruption for therapeutic purpose were conducted for the patients with *β*-thalassemia and sickle-cell anemia, therapy named as CTX001. It involves CRISPR-mediated disruption of *BCL*_*11*_ *A* gene in autologous progenitor and hematopoietic stem cells collected from peripheral blood (Basak et al., 2015; Basak and Sankaran, 2016). The first trial with *in vivo* delivery of CRISPR/Cas9 were conducted in leber congenital amaurosis patients in which a non-functional protein is produced due to the intronic IVS26 mutation, a therapy named as EDIT-101 (Maeder et al., 2019). Similarly, 32-base deletion was conducted in CCR5 gene (CCR5Δ32) of human embryos by using CRISPR/Cas9 and both embryos were implanted back into their mother (Cohen, 2019; Greely, 2019). In current state, germline gene editing is ethically unfavorable unless safety data of ongoing somatic CRISPR-based therapy clinical trials is obtained.

Recently, a novel severe acute respiratory syndrome coronavirus 2 (SARS-CoV-2) caused a global pandemic, coronavirus disease 2019 (COVID-19) (Sironi et al., 2020). The timely detection of SARS-CoV-2 and its cure is very important to prevent the death of the patients. A CRISPR Cas12-based assay has been developed to detect COVID-19 with 95% accuracy and turnaround time of around 40 min, named as SARS-CoV-2 DETECTR. The assay involves isothermal amplification of reverse transcribed RNA of SARS-CoV-2. Cas12 and guide RNAs against nucleoprotein and envelop genes are targeted and their cleavage is visualized by fluorescent reporter system (Broughton et al., 2020). In addition to detection, CRISPR may also provide therapeutic potential for COVID-19 patients. The PAC-MAN (Prophylactic Antiviral CRISPR in huMAN cells) utilizes *Ruminococcus flavefaciens* derived VI-D CRISPR-Cas13d variant which can degrade the SARS-CoV-2 RNA by simultaneously targeting multiple regions (Abbott et al., 2020). With these advancements, CRISPR/Cas machinery may serve as a virus-battling system during this pandemic.

## 7 Concluding Remarks

Not long ago, genome editing in humans was considered a hypothetical idea, but the CRISPR tool has provided a hopeful platform to achieve it. The CRISPR/Cas system is an emerging biotechnological tool for genome editing. In the presence of other competitive tools, such as like ZFNs and TALEN, what makes CRISPR better? The answer lies in the high cost-effectiveness, less laborious, specificity, and efficient compared to others. Since the application of CRISPR/Cas system into the genome editing, various experimental improvements have been achieved to enhance its specificity and efficiency (Table 1). Despite the swift progress in the CRISPR field, there are many fundamental unanswered mechanistic questions regarding spacer acquisition and discriminating between self and non-self among various CRISPR subtypes. The mechanism of crRNA biogenesis and interference is relatively well understood for certain subtypes, such as type I-E and type II-A. However, Type IV, V, and VI needs to be characterized further as some of them show mechanisms different from the traditional systems (Makarova and Koonin, 2015). In addition, many subtypes with possibly novel working mechanisms need to be discovered to enhance biotechnological application of the system.

**TABLE 1:** Summary to enhance the efficiency of CRISPR-mediated genome and epigenome editing.

Sr. no	Recommendation/Strategy
1	GC content of designed sgRNA must range from >30% to <70%
2	Target promoter region rather than exon or intron for gene disruption (if possible)
3	Prefer purine-rich spacer sequences (if possible)
4	Existence of secondary structure in sgRNA improves its processing and genome-editing capability
5	Truncating gRNA or adding extra guanines at its 5′end increases its specificity
6	Stabilize the gRNA with G-quadruplexe structure
7	Eliminate seed regions with UUU sequence
8	Chemically modify the gRNA
9	Substitute one of the nucleotides in the continuous stretch of four to six
10	Avoid constitutively higher expression level of sgRNA and Cas9 to prevent off-targeting
11	Select appropriate method to deliver CRISPR components
12	Avoid targeting heterochromatin region (if possible)
13	Inhibit NHEJ or stimulate HDR to increase the knock-in efficiency of transgene
14	Select appropriate type of donor-template and DNA-repair pathway
15	Use SpCas9_MT_-pDBD, paired nickase-Cas9 or dCas9-FokI to increase specificity
16	Prefer PAM-out orientation over PAM-in and D10A mutant over H840A
17	Use dead-guide or dead-Cas9 for epigenome editing
18	Recruit multiple activators by using modified guide and dCas9; combining dCas9 system with multipeptide array like SunTag; or using tripartite system to increase the efficiency of CRISPR activation
19	Modify dCas9 or sgRNA to recruit epigenetic modifiers at the target site

There are still challenges related to off-targeting and less efficiency caused by Cas9 in the clinical and *in vitro* research venue. Moreover, dominating HDR over NHEJ to enhance the process of homology-based knock-in of transgenes remains a challenge (Chu et al., 2015; Maruyama et al., 2015). The Cas endonuclease can be delivered in various ways (Supplementary Table S3) in the form of DNA, mRNA, or protein to maximize the output in clinical applications; however, selecting an appropriate delivery method for a particular form is still a challenge (Lin et al., 2014a; Gori et al., 2015; Howes and Schofield, 2015; Zuris et al., 2015). Some of the advanced applications, such as CRISPR-enChIP, also face challenges like off-targeting (O'Geen et al., 2015). Due to its capability of genome editing in germlines, various social and ethical issues must be considered while editing not only in humans but also in other organisms (Rodriguez, 2016).

The precise and efficient genome editing are the main attributes of CRISPR/Cas technology; however, these might not remain same from cell to cell; cell to organism; or organism to organism. Based on research, various parameters and modifications have a significant role in improving the specificity and efficiency of genome editing. Briefly, number and nature of sgRNA; expression level and delivery of CRISPR components; features of target sequence; host DNA-repair pathways; and modified versions of Cas9 and guide RNA must be kept under consideration.

Other aspects of the CRISPR/Cas system do not fit the scope of this review. Along with providing adaptive-immunity (Figure 1), it also regulates genomic evolution, DNA repair, and group behavior (Westra et al., 2014; Ratner et al., 2015). The spatiotemporal regulation of the CRISPR/Cas system in response to the stress signal and phage infection requires further study to understand the complete mechanism (Bondy-Denomy and Davidson, 2014; Garrett et al., 2015; Patterson et al., 2015). Another active research area has been opened after identifying phage evasion of CRISPR-mediated immunity by various mechanisms, including mutational escape, anti-CRISPR proteins, and DNA modification (Deveau et al., 2008; Bondy-Denomy et al., 2013; Bondy-Denomy et al., 2015; Bryson et al., 2015; Paez-Espino et al., 2015). Further exploration of various organisms promises to discover new technologies like that of the CRISPR/Cas system.

## Glossary

CRISPR: clustered regularly interspaced short palindromic repeats
Cas: CRISPR-associated protein
HR: homologous recombination
DSBs: double-strand breaks
indels: insertion and deletions
NHEJ: non-homologous end joining
ZFNs: zinc finger nucleases
TALENs: transcription activator-like effector nucleases
PAM: protospacer adjacent motif
Cascade: CRISPR associated complex for antiviral defense
tracrRNA: *trans*-activating crRNA
sgRNA: single guide RNA
HDR: homology directed repair
AV: adenovirus vector
AAV: adeno-associated vector
RNP: ribonucleoprotein
ssDNA: single stranded DNA
MMEJ: microhomology-mediated end joining
ssODNs: single-stranded oligodeoxyribonucleotides
RFNs: RNA-guided FokI-dCas9 nucleases
trugRNAs: truncated gRNAs
pDBD: programmable DNA-binding domain
HiFi Cas9: high-fidelity Cas9 variant
PID: PAM-interacting domain
St3Cas9: *Streptococcus thermophiles* CRISPR-3 Cas9
dRNA: dead guide
dCas9: dead or deactivated Cas9
CRISPRa: CRISPR Activation
MCPs: MS2 bacteriophage coat proteins
scFv: single-chain variable fragment
CXCR4: chemokine (C-X-C motif) receptor 4
CDKN1B: cyclin-dependent kinase inhibitor 1B
Nm dCas9: *Neisseria meningitidis* dcas9
Sp dCas9: *Streptococcus pyogenes* dCas9
FPs: fluorescent proteins
enCHIP: engineered DNA-binding molecule-mediated ChIP
scRNA: scaffold RNA
RBPs: RNA-binding proteins
RNAi: RNA interference
GeCKO: genome-scale CRISPR-Cas9 knockout
CRISPRi: CRISPR-mediated repression
CFTR: cystic fibrosis transmembrane conductor receptor
LTR: long terminal repeats
TCR: T cell receptor
SARS-CoV-2: severe acute respiratory syndrome coronavirus 2
COVID-19: coronavirus disease 2019
PAC-MAN: Prophylactic Antiviral CRISPR in huMAN
