# Supplementary material for: CRISPR/Cas System and Factors Affecting Its Precision and Efficiency
Source: Front Cell Dev Biol. 2021 Nov 24;9:761709. doi: 10.3389/fcell.2021.761709 (PMC8652214; doi:10.3389/fcell.2021.761709)
Supplement: Supplementary file 1 [file Table1.DOCX]

Supplementary Material

# Supplementary Tables

**Supplementary Table 1.** Orthologues and their engineered variants from the class II CRISPR/Cas system.

| **a) Properties of representative orthologues from various types** | | | | | |
| --- | --- | --- | --- | --- | --- |
| **Species and enzyme** | **Orthologue (system type)** | **Length (aa)** | **Target region length (nt)** | **PAM sequence (5’ to 3’)** | **Reference** |
| *Staphylococcus*  *aureus* Cas9 | SaCas9 (II) | 1,053 | 20-24 | NNGRRT | ([Ran et al., 2015](#_ENREF_54)) |
| *Streptococcus*  *thermophilus*1  Cas9 | St1Cas9 (II) | 1,122 | 19-20 | NNAGAAW | ([Cong et al., 2013](#_ENREF_9); [Fujii et al., 2016](#_ENREF_17)) |
| *Streptococcus*  *thermophilus*3  Cas9 | St3Cas9 (II) | 1,393 | 19 | NGGNG | ([Xu et al., 2015b](#_ENREF_71); [Müller et al., 2016](#_ENREF_43)) |
| *Neisseria meningitidis* Cas9 | NmCas9 (II) | 1,109 | 23,24 | NNNNGATT | ([Lee et al., 2016](#_ENREF_33)) |
| *Francisella novicida* Cas9 | FnCas9 (II) | 1,629 | 22 | NGG | ([Hirano et al., 2016](#_ENREF_21)) |
| *Treponema denticola* Cas9 | TdCas9 (II) | 1,423 | 20 | NAAAAN | ([Esvelt et al., 2013](#_ENREF_14)) |
| *Campylobacter*  *jejuni* Cas9 | CjCas9 (II) | 984 | 22 | NNNNACAC  or NNNNRYAC | ([Kim et al., 2017a](#_ENREF_26)) |
| *Acidaminococcus sp.*  Cas12a (Cpf1) | AsCas12a or AsCpf1 (V-A) | 1,307 | 23,24 | TTTV | ([Watkins-Chow et al., 2017](#_ENREF_67)) |
| *Lachnospiraceae sp.*  Cas12a (Cpf1) | LbCas12a or LbCpf1 (V-A) | 1,228 | 23,24 | TTTV | ([Kim et al., 2017b](#_ENREF_27)) |
| *Francisella novicida* Cpf1 (FnCpf1) | FnCpf1 (V-A) | 1,300 | 23-25 | TTN | ([Zetsche et al., 2015](#_ENREF_74)) |
| *Alicyclobacillus acidoterrestris* Cas12b (C2c1) | AacC2c1 (V-B) | 1,129 | 20 | TTN | ([Shmakov et al., 2015](#_ENREF_58)) |
| *Bacillus hisashii,* Cas12b (C2c1) | BhCas12b (V-B) | 1,108 | 20 | ATTN | ([Strecker et al., 2019](#_ENREF_61)) |
| *Leptotrichia*  *buccalis* Cas13a  (C2c2) | LbuCas13a or LbuC2c2 (VI-A) | 1,159 | 20-28 | Non-G protospacer flanking site (PFS) | ([East-Seletsky et al., 2016](#_ENREF_12)) |
| *Leptotrichia*  *shahii* Cas13a  (C2c2) | LshCas13a or LshC2c2 (VI-A) | 1,389 | 20-28 |  | ([Abudayyeh et al., 2016](#_ENREF_1)) |
| **b) Representative engineered variants of various orthologues** | | | | | |
| **CRISPR component** | **Engineered type** | **Modification** | | **Importance** | **Reference** |
| SpCas9 | Nickase-SpCas9 | D10A or H840A point mutations in the nuclease domain of SpCas9 | | Enhanced Specificity | ([Jinek et al., 2012](#_ENREF_25); [Ran et al., 2013](#_ENREF_55)) |
|  | dSpCas9 | Inactivation of both nuclease domains by mutations (D10A and H840A) | | Epigenome editing | ([Qi et al., 2013](#_ENREF_52)) |
|  | dSpCas9-Dnmt3a and –Tet1 | Fusion of dCas9 with Dnmt3a or Tet1, respectively | | Regulation of CpG methylation | ([Liu et al., 2016](#_ENREF_37)) |
|  | eSpCas9 | Substituting three codon within non-targeted DNA strand groove of Cas9 | | Increased stringency-mediated enhanced specificity | ([Slaymaker et al., 2016](#_ENREF_59)) |
|  | SpCas9-HF | Substituting four alanine residues interacting with phosphate backbone of the targeted DNA strand | | Improved specificity without much variation in efficiency | ([Kleinstiver et al., 2016](#_ENREF_28)) |
|  | Split‐SpCas9 | Expression of α-helical and nuclease lobes as separate polypeptides | | More regulated editing | ([Wright et al., 2015](#_ENREF_69)) |
|  | SpCas9‐cytidine deaminase | Fusion of dCas9 and cytidine deaminase | | Programmed, direct and permanent substitution of DNA base without breaking target dsDNA | ([Komor et al., 2016](#_ENREF_31); [Nishida et al., 2016](#_ENREF_45)) |
|  | VQR-SpCas9 | D1135V/R1335Q/T1337R | | Broad target range because of alternate PAM recognition | ([Kleinstiver et al., 2015b](#_ENREF_30)) |
|  | EQR-SpCas9 | D1135E/R1335Q/T1337R | |  |  |
|  | VRER-SpCas9 | D1135V/G1218R/R1335E/T1337R | |  |  |
|  | QQR1-SpCas9 | QQR1 | |  | ([Anders et al., 2016](#_ENREF_2)) |
|  | D1135E-SpCas9 | D1135E | | improved PAM recognition and specificity | ([Kleinstiver et al., 2016](#_ENREF_28)) |
|  | St3-SpCas9 | St3Cas9 with PAM-interacting domain (PID) of SpCas9 | | Alternate PAM recognition | ([Nishimasu et al., 2014](#_ENREF_46)) |
| SaCas9 | eSaCas9 | Substituting four codons within non-targeted DNA strand groove of Cas9 | | Enhanced specificity because of increased stringency between RNA-DNA interaction | ([Slaymaker et al., 2016](#_ENREF_59)) |
|  | Nickase-SaCas9 | D10A or N580A point mutations in the nuclease domain of SaCas9 | | Enhanced specificity | ([Friedland et al., 2015](#_ENREF_16)) |
|  | dSaCas9 | Inactivation of both nuclease domains by mutations (D10A and N580A) | | Epigenome editing | ([Ran et al., 2015](#_ENREF_54)) |
|  | KKH variant | E782K/N968K/R1015H | | Altered PAM (NNNRRT) | ([Kleinstiver et al., 2015a](#_ENREF_29)) |
| St1Cas9 | dSt1Cas9 | Inactivation of nuclease domains | | Epigenome editing (however lesser efficient) | ([Lian et al., 2017](#_ENREF_36)) |
| St3Cas9 | dSt3Cas9 | Inactivation of nuclease domains (D10A and N870A) | | Epigenome editing | ([Müller et al., 2016](#_ENREF_43)) |
| NmCas9 | dNmCas9 | Inactivation of nuclease domains (D16A, D587A, H588A and N611A) | | Epigenome editing | ([Bolukbasi et al., 2018](#_ENREF_4)) |
| FnCas9 | RHA variant | E1369R/E1449H/R1556A | | Altered PAM (YG) | ([Hirano et al., 2016](#_ENREF_21)) |
| CjCas9 | Nickase Cjcas9 | Inactivation of nuclease domains (D8A, H559A or ΔHNH) | | More specific than wild-type | ([Yamada et al., 2017](#_ENREF_72)) |
|  | Cjdcas9 | Inactivation of nuclease domains (D8A and H559A) | | Epigenome editing | ([Chen et al., 2017](#_ENREF_7)) |
| AsCpf1 | dAsCpf1 | Inactivation of nuclease domain (D908A) | | Epigenome editing | ([Liu et al., 2017](#_ENREF_38)) |
|  | RR variant | S542R/K607R | | Altered PAM (TYCV) | ([Gao et al., 2017](#_ENREF_20)) |
|  | RVR variant | S542R/K548V/N552R | | Altered PAM (TATV) |  |
| LbCpf1 | dLbCpf1 | Inactivation of nuclease domains | | Epigenome editing | ([Lian et al., 2017](#_ENREF_36)) |
|  | RR variant | G532R/K595R | | Altered PAM (TYCV) | ([Gao et al., 2017](#_ENREF_20)) |
|  | RVR variant | G532R/K538V/Y542R | | Altered PAM (TATV) |  |
| BhCas12b | BhCas12b v4 | K846R/S893R/E837G | | dsDNA cleavage at 37°C | ([Strecker et al., 2019](#_ENREF_61)) |
| LbCas13a or LbC2c2 | dLbCas13a or dLbC2c2 | Inactivation of HEPN nuclease domains (R472A, H477A, R1048A, and H1053) | | Epigenome editing | ([East-Seletsky et al., 2016](#_ENREF_12)) |
| LshCas13a or LshC2c2 | dLshCas13a or dLshC2c2 | Inactivation of HEPN nuclease domains (R597A, H602A, R1278A, and H1283A) | | Epigenome editing | ([Abudayyeh et al., 2016](#_ENREF_1)) |

**Supplementary Table 2.** Online tools to predict the most efficient gRNA.

| **Tool Name** | **URL address** | **Enzyme** | **gRNA activity prediction** | **Maximum number of supported mismatches** | **Available PAMs** | **Reference** |
| --- | --- | --- | --- | --- | --- | --- |
| CRISPOR | <http://crispor.tefor.net/> | SpCas9, SaCas9, xCas9, NmCas9, CjCas9, Cas12a | Yes | 4 | NGG, NGA, NGCG, NNAGAA, NGGNG, NNGRRT, NNNRRT, NNNNGMTT, NNNNACA, TTTN | ([Farboud and Meyer, 2015](#_ENREF_15)) |
| crisprscan | <http://www.crisprscan.org/> | SpCas9, LbCpf1, AsCpf | Yes | 2 | NGG, TTTV, TTTN | ([Moreno-Mateos et al., 2015](#_ENREF_41)) |
| evaluateCRISPR | <http://www.flyrnai.org/evaluateCrispr/> | - | Yes | 5 | - | ([Housden et al., 2015](#_ENREF_22)) |
| Sequence Scan for CRISPR (SSC) | <http://cistrome.org/SSC/> | SpCas9 | Yes | - | NGG | ([Xu et al., 2015a](#_ENREF_70)) |
| sgrna-design | <http://portals.broadinstitute.org/gpp/public/analysis-tools/sgrna-design> | SpCas9, SaCas9 | Yes | 1 | NGG, NNGRR | ([Fusi et al., 2015](#_ENREF_18)) |
| Benchling CRISPR gRNA Design | https://benchling.com/crispr | Cas9 | Yes | 4 | Customizable | ([Ran et al., 2013](#_ENREF_55); [Doench et al., 2014](#_ENREF_11)) |
| Breaking-Cas | http://bioinfogp.cnb.csic.es/tools/breakingcas/ | SpCas9, SaCas9, xCas9, AsCpf1 | Yes | 4 | Customizable | ([Oliveros et al., 2016](#_ENREF_48)) |
| Cas-OFFinder | http://www.rgenome.net/cas-offinder/ | SpCas9, StCas9, NmCas9, SaCas9, CjCas9, AsCpf1, FnCpf1 | Yes | 0-10 | Customizable | ([Oliveros et al., 2016](#_ENREF_48)) |
| CCTop | https://crispr.cos.uni-heidelberg.de/ | SpCas9, SaCas9, NmCas9, StCas9, TdCas9, AsCpf1 | Yes | 0-5 | NGG, NRG, NNGRRT, NNNNGATT, NNAGAAW, NAAAAC, TTTN | ([Stemmer et al., 2015](#_ENREF_60)) |
| CHOPCHOP v2 | http://chopchop.cbu.uib.no/ | SpCas9, StCas9, NmCas9, SaCas9, AsCpf1, FnCpf1 | Yes | 0-3 | customizable | ([Labun et al., 2016](#_ENREF_32)) |
| CRISPRdirect | http://crispr.dbcls.jp/ | SpCas9 | Yes | Any | NGG, NRG | ([Naito et al., 2014](#_ENREF_44)) |
| ATUM gRNA Design Tool | https://www.atum.bio/eCommerce/cas9/input | SpCas9 | Yes | 0-10 | NRG | https://www.atum.bio |
| CRISPR Design Tool | https://dharmacon.horizondiscovery.com/gene-editing/crispr-cas9/crispr-design-tool/ | SpCas9 | No | 8 | NRG | https://dharmacon.horizondiscovery.com |
| CRISPRseek | http://www.bioconductor.org/packages/release/bioc/html/CRISPRseek.html | Customizable | Yes | Any | Customizable | ([Zhu et al., 2014](#_ENREF_76)) |
| WU-CRISPR | <http://crispr.wustl.edu/> | SpCas9 | Yes | - | NGG | ([Wong et al., 2015](#_ENREF_68)) |
| DESKGEN | https://www.deskgen.com/landing/ | Customizable | Yes | Any | Customizable | <https://www.deskgen.com> |
| Geneious CRISPR Site Finder | https://www.geneious.com/ | Customizable | Yes | Any | Customizable | ([Doench et al., 2014](#_ENREF_11)) |
| GT-Scan | https://gt-scan.csiro.au/ | Customizable | Yes | 0-3 | Customizable | ([O’Brien and Bailey, 2014](#_ENREF_47)) |
| Off-Spotter | https://cm.jefferson.edu/Off-Spotter/ | SpCas9, CjCas9, SaCas9 | Customizable | 0-5 | NGG, NAG, NNNNACA, NNGRRT | ([Pliatsika and Rigoutsos, 2015](#_ENREF_50)) |
| Synthego Design Tool | https://design.synthego.com/#/ | SpCas9 | Yes | 3 | NGG | <https://www.synthego.com> |

**Supplementary Table 3.** Comparison of various methods to deliver CRISPR/Cas components.

| **a) Viral delivery methods** | | | | | | | | | |
| --- | --- | --- | --- | --- | --- | --- | --- | --- | --- |
| **Name** | **Composition** | **Packaging Capacity (kb)** | **Cargo** | **Non-Dividing Cell Transduction** | **Genome Integration** | **Main Pros** | | **Main Cons** | **Reference** |
| Retrovirus | RNA, enveloped | 8 | DNA | No | Yes | Persistant transfer in dividing cells | | Oncogenic | ([Malina et al., 2013](#_ENREF_40)) |
| Lentivirus | RNA, enveloped | 8 | DNA | Yes | Yes | Persistant transfer in most tissues | | Oncogenic | ([Roehm et al., 2016](#_ENREF_56); [Zhang et al., 2016](#_ENREF_75)) |
| Adenovirus (AV) | dsDNA, non-enveloped | 8-30 | DNA | Yes | No | Efficient transduction | | Capsid based immune response | ([Maggio et al., 2016](#_ENREF_39); [Voets et al., 2017](#_ENREF_65)) |
| Adeno-associated Virus (AAV) | ssDNA, non-enveloped | <5 | DNA | Yes | No | Non-infalmmatory and pathogenic | | Small packaging capacity | ([Tabebordbar et al., 2016](#_ENREF_64); [Wang et al., 2018](#_ENREF_66)) |
| **b) Non-viral delivery methods** | | | | | | | | | |
| **Name** | **Composition** | **Packaging Capacity** | **Cargo** | | **Pros** | | **Cons** | | **Reference** |
| Nucleofection, electroporation | Electric shock | nM | DNA, mRNA | | Efficient than lipofection | | *In vitro* only, disruption of cellular barriers | | ([Chen et al., 2016](#_ENREF_8); [Brunetti et al., 2018](#_ENREF_5)) |
| Microinjection | Needle based | nM | DNA, mRNA, protein | | Efficient targeted delivery, *in vitro* and *in vivo* | | Difficult to perform | | ([Gagnon et al., 2014](#_ENREF_19); [Elaswad et al., 2018](#_ENREF_13); [Poirier et al., 2019](#_ENREF_51); [Chambers et al., 2020](#_ENREF_6)) |
| Hydrodynamic | Compressed injection | nM | DNA, protein | | Easy, economical, virus-free | | Tissue injury, non-specific | | ([Hubner et al., 2018](#_ENREF_24); [Pankowicz et al., 2018](#_ENREF_49)) |
| Liposomes, lipoplexes, polyplexes | Lipid, polyethenimine (PEI), poly(L-lysine) (PLL | nM | DNA, mRNA, protein | | Simple, economical, virus-free | | Endosomal degradation, harder nuclear delivery | | ([Li et al., 2018](#_ENREF_35); [Ryu et al., 2018](#_ENREF_57)) |
| Cell penetrating peptides (CPPs) | Positive/hydrophobic amino acids | nM | Protein | | Intact and virus-free delivery | | Variable efficiency, low cytosolic distribution | | ([Ramakrishna et al., 2014](#_ENREF_53); [Axford et al., 2017](#_ENREF_3)) |
| Gold nanoparticles | Gold particles coated with cationic arginine | nM | Protein | | Non-viral, inert, non-immunogenic | | Immunogenic inflammatory response | | ([Lee et al., 2017](#_ENREF_34); [Mout et al., 2017](#_ENREF_42)) |
| Magnetofection | Magnetic iron oxide core coated with PEI | μg-mg | DNA | | nanoscale size, high surface area, stability and biocompatibility | | Little toxic | | ([Hryhorowicz et al., 2018](#_ENREF_23)) |
| Graphene oxide nanoparticle | graphene oxide (GO)-polyethylene glycol (PEG)-polyethylenimine (PEI) | nM | RNP | | good  stability, excellent biocompatibility, and low toxicity, higher specific surface area, thereby effectively enhancing the payload capacity | | cytotoxicity | | ([Yue et al., 2018](#_ENREF_73)) |
| DNA nanoclew | Spherical DNA | nM | Protein | | Non-viral | | Modifications require for increased efficiency | | ([Sun et al., 2014](#_ENREF_63); [Sun et al., 2015](#_ENREF_62)) |
| iTOP | Hyperosmolality and transduction compound | nM | Protein | | Non-viral, efficient | | In-vitro only, non-specific | | ([D’Astolfo et al., 2015](#_ENREF_10)) |

# References

Abudayyeh, O.O., Gootenberg, J.S., Konermann, S., Joung, J., Slaymaker, I.M., Cox, D.B., et al. (2016). C2c2 is a single-component programmable RNA-guided RNA-targeting CRISPR effector. *Science* 353(6299)**,** aaf5573.

Anders, C., Bargsten, K., and Jinek, M. (2016). Structural plasticity of PAM recognition by engineered variants of the RNA-guided endonuclease Cas9. *Molecular cell* 61(6)**,** 895-902.

Axford, D.S., Morris, D.P., and McMurry, J.L. (2017). Cell penetrating peptide-mediated nuclear delivery of Cas9 to enhance the utility of CRISPR/Cas genome editing. *The FASEB Journal* 31(1_supplement)**,** 909.904-909.904.

Bolukbasi, M.F., Liu, P., Luk, K., Kwok, S.F., Gupta, A., Amrani, N., et al. (2018). Orthogonal Cas9–Cas9 chimeras provide a versatile platform for genome editing. *Nature communications* 9(1)**,** 4856.

Brunetti, L., Gundry, M.C., Kitano, A., Nakada, D., and Goodell, M.A. (2018). Highly Efficient Gene Disruption of Murine and Human Hematopoietic Progenitor Cells by CRISPR/Cas9. *Journal of visualized experiments: JoVE* (134).

Chambers, B.E., Clark, E.G., Gatz, A.E., and Wingert, R.A. (2020). Kctd15 regulates nephron segment development by repressing Tfap2a activity. *Development* 147(23)**,** dev191973.

Chen, F., Ding, X., Feng, Y., Seebeck, T., Jiang, Y., and Davis, G.D. (2017). Targeted activation of diverse CRISPR-Cas systems for mammalian genome editing via proximal CRISPR targeting. *Nature communications* 8**,** 14958.

Chen, S., Lee, B., Lee, A.Y.-F., Modzelewski, A.J., and He, L. (2016). Highly efficient mouse genome editing by CRISPR ribonucleoprotein electroporation of zygotes. *Journal of Biological Chemistry***,** jbc. M116. 733154.

Cong, L., Ran, F.A., Cox, D., Lin, S., Barretto, R., Habib, N., et al. (2013). Multiplex genome engineering using CRISPR/Cas systems. *Science***,** 1231143.

D’Astolfo, D.S., Pagliero, R.J., Pras, A., Karthaus, W.R., Clevers, H., Prasad, V., et al. (2015). Efficient intracellular delivery of native proteins. *Cell* 161(3)**,** 674-690.

Doench, J.G., Hartenian, E., Graham, D.B., Tothova, Z., Hegde, M., Smith, I., et al. (2014). Rational design of highly active sgRNAs for CRISPR-Cas9–mediated gene inactivation. *Nature biotechnology* 32(12)**,** 1262.

East-Seletsky, A., O’Connell, M.R., Knight, S.C., Burstein, D., Cate, J.H., Tjian, R., et al. (2016). Two distinct RNase activities of CRISPR-C2c2 enable guide-RNA processing and RNA detection. *Nature* 538(7624)**,** 270.

Elaswad, A., Khalil, K., Cline, D., Page-McCaw, P., Chen, W., Michel, M., et al. (2018). Microinjection of CRISPR/Cas9 protein into channel catfish, Ictalurus punctatus, embryos for gene editing. *JoVE (Journal of Visualized Experiments)* (131)**,** e56275.

Esvelt, K.M., Mali, P., Braff, J.L., Moosburner, M., Yaung, S.J., and Church, G.M. (2013). Orthogonal Cas9 proteins for RNA-guided gene regulation and editing. *Nature methods* 10(11)**,** 1116-1121.

Farboud, B., and Meyer, B.J. (2015). Dramatic enhancement of genome editing by CRISPR/Cas9 through improved guide RNA design. *Genetics* 199(4)**,** 959-971.

Friedland, A.E., Baral, R., Singhal, P., Loveluck, K., Shen, S., Sanchez, M., et al. (2015). Characterization of Staphylococcus aureus Cas9: a smaller Cas9 for all-in-one adeno-associated virus delivery and paired nickase applications. *Genome biology* 16(1)**,** 257.

Fujii, W., Kakuta, S., Yoshioka, S., Kyuwa, S., Sugiura, K., and Naito, K. (2016). Zygote-mediated generation of genome-modified mice using Streptococcus thermophilus 1-derived CRISPR/Cas system. *Biochemical and biophysical research communications* 477(3)**,** 473-476.

Fusi, N., Smith, I., Doench, J., and Listgarten, J. (2015). In silico predictive modeling of CRISPR/Cas9 guide efficiency. *bioRxiv***,** 021568.

Gagnon, J.A., Valen, E., Thyme, S.B., Huang, P., Ahkmetova, L., Pauli, A., et al. (2014). Efficient mutagenesis by Cas9 protein-mediated oligonucleotide insertion and large-scale assessment of single-guide RNAs. *PloS one* 9(5)**,** e98186.

Gao, L., Cox, D.B., Yan, W.X., Manteiga, J.C., Schneider, M.W., Yamano, T., et al. (2017). Engineered Cpf1 variants with altered PAM specificities. *Nature biotechnology* 35(8)**,** 789.

Hirano, H., Gootenberg, J.S., Horii, T., Abudayyeh, O.O., Kimura, M., Hsu, P.D., et al. (2016). Structure and engineering of Francisella novicida Cas9. *Cell* 164(5)**,** 950-961.

Housden, B.E., Valvezan, A.J., Kelley, C., Sopko, R., Hu, Y., Roesel, C., et al. (2015). Identification of potential drug targets for tuberous sclerosis complex by synthetic screens combining CRISPR-based knockouts with RNAi. *Science signaling* 8(393)**,** rs9.

Hryhorowicz, M., Grześkowiak, B., Mazurkiewicz, N., Śledziński, P., Lipiński, D., and Słomski, R. (2018). Improved Delivery of CRISPR/Cas9 System Using Magnetic Nanoparticles into Porcine Fibroblast. *Molecular biotechnology***,** 1-8.

Hubner, E.K., Lechler, C., Rösner, T.N., Kohnke-Ertel, B., Schmid, R.M., and Ehmer, U. (2018). Constitutive and Inducible Systems for Genetic In Vivo Modification of Mouse Hepatocytes Using Hydrodynamic Tail Vein Injection. *JoVE (Journal of Visualized Experiments)* (132)**,** e56613.

Jinek, M., Chylinski, K., Fonfara, I., Hauer, M., Doudna, J.A., and Charpentier, E. (2012). A programmable dual-RNA–guided DNA endonuclease in adaptive bacterial immunity. *Science***,** 1225829.

Kim, E., Koo, T., Park, S.W., Kim, D., Kim, K., Cho, H.-Y., et al. (2017a). In vivo genome editing with a small Cas9 orthologue derived from Campylobacter jejuni. *Nature Communications* 8**,** 14500.

Kim, H.K., Song, M., Lee, J., Menon, A.V., Jung, S., Kang, Y.-M., et al. (2017b). In vivo high-throughput profiling of CRISPR–Cpf1 activity. *Nature methods* 14(2)**,** 153.

Kleinstiver, B.P., Pattanayak, V., Prew, M.S., Tsai, S.Q., Nguyen, N.T., Zheng, Z., et al. (2016). High-fidelity CRISPR–Cas9 nucleases with no detectable genome-wide off-target effects. *Nature* 529(7587)**,** 490-495.

Kleinstiver, B.P., Prew, M.S., Tsai, S.Q., Nguyen, N.T., Topkar, V.V., Zheng, Z., et al. (2015a). Broadening the targeting range of Staphylococcus aureus CRISPR-Cas9 by modifying PAM recognition. *Nature biotechnology* 33(12)**,** 1293.

Kleinstiver, B.P., Prew, M.S., Tsai, S.Q., Topkar, V.V., Nguyen, N.T., Zheng, Z., et al. (2015b). Engineered CRISPR-Cas9 nucleases with altered PAM specificities. *Nature* 523(7561)**,** 481.

Komor, A.C., Kim, Y.B., Packer, M.S., Zuris, J.A., and Liu, D.R. (2016). Programmable editing of a target base in genomic DNA without double-stranded DNA cleavage. *Nature* 533(7603)**,** 420.

Labun, K., Montague, T.G., Gagnon, J.A., Thyme, S.B., and Valen, E. (2016). CHOPCHOP v2: a web tool for the next generation of CRISPR genome engineering. *Nucleic acids research* 44(W1)**,** W272-W276.

Lee, C.M., Cradick, T.J., and Bao, G. (2016). The Neisseria meningitidis CRISPR-Cas9 system enables specific genome editing in mammalian cells. *Molecular Therapy* 24(3)**,** 645-654.

Lee, K., Conboy, M., Park, H.M., Jiang, F., Kim, H.J., Dewitt, M.A., et al. (2017). Nanoparticle delivery of Cas9 ribonucleoprotein and donor DNA in vivo induces homology-directed DNA repair. *Nature biomedical engineering* 1(11)**,** 889.

Li, X., Aghaamoo, M., Liu, S., Lee, D.H., and Lee, A.P. (2018). Lipoplex‐Mediated Single‐Cell Transfection via Droplet Microfluidics. *Small* 14(40)**,** 1802055.

Lian, J., HamediRad, M., Hu, S., and Zhao, H. (2017). Combinatorial metabolic engineering using an orthogonal tri-functional CRISPR system. *Nature communications* 8(1)**,** 1688.

Liu, X.S., Wu, H., Ji, X., Stelzer, Y., Wu, X., Czauderna, S., et al. (2016). Editing DNA methylation in the mammalian genome. *Cell* 167(1)**,** 233-247. e217.

Liu, Y., Han, J., Chen, Z., Wu, H., Dong, H., and Nie, G. (2017). Engineering cell signaling using tunable CRISPR–Cpf1-based transcription factors. *Nature communications* 8(1)**,** 2095.

Maggio, I., Stefanucci, L., Janssen, J.M., Liu, J., Chen, X., Mouly, V., et al. (2016). Selection-free gene repair after adenoviral vector transduction of designer nucleases: rescue of dystrophin synthesis in DMD muscle cell populations. *Nucleic acids research* 44(3)**,** 1449-1470.

Malina, A., Mills, J.R., Cencic, R., Yan, Y., Fraser, J., Schippers, L.M., et al. (2013). Repurposing CRISPR/Cas9 for in situ functional assays. *Genes & development* 27(23)**,** 2602-2614.

Moreno-Mateos, M.A., Vejnar, C.E., Beaudoin, J.-D., Fernandez, J.P., Mis, E.K., Khokha, M.K., et al. (2015). CRISPRscan: designing highly efficient sgRNAs for CRISPR-Cas9 targeting in vivo. *Nature methods* 12(10)**,** 982-988.

Mout, R., Ray, M., Yesilbag Tonga, G., Lee, Y.-W., Tay, T., Sasaki, K., et al. (2017). Direct cytosolic delivery of CRISPR/Cas9-ribonucleoprotein for efficient gene editing. *ACS nano* 11(3)**,** 2452-2458.

Müller, M., Lee, C.M., Gasiunas, G., Davis, T.H., Cradick, T.J., Siksnys, V., et al. (2016). Streptococcus thermophilus CRISPR-Cas9 systems enable specific editing of the human genome. *Molecular Therapy* 24(3)**,** 636-644.

Naito, Y., Hino, K., Bono, H., and Ui-Tei, K. (2014). CRISPRdirect: software for designing CRISPR/Cas guide RNA with reduced off-target sites. *Bioinformatics* 31(7)**,** 1120-1123.

Nishida, K., Arazoe, T., Yachie, N., Banno, S., Kakimoto, M., Tabata, M., et al. (2016). Targeted nucleotide editing using hybrid prokaryotic and vertebrate adaptive immune systems. *Science* 353(6305)**,** aaf8729.

Nishimasu, H., Ran, F.A., Hsu, P.D., Konermann, S., Shehata, S.I., Dohmae, N., et al. (2014). Crystal structure of Cas9 in complex with guide RNA and target DNA. *Cell* 156(5)**,** 935-949.

O’Brien, A., and Bailey, T.L. (2014). GT-Scan: identifying unique genomic targets. *Bioinformatics* 30(18)**,** 2673-2675.

Oliveros, J.C., Franch, M., Tabas-Madrid, D., San-León, D., Montoliu, L., Cubas, P., et al. (2016). Breaking-Cas—interactive design of guide RNAs for CRISPR-Cas experiments for ENSEMBL genomes. *Nucleic acids research* 44(W1)**,** W267-W271.

Pankowicz, F.P., Barzi, M., Kim, K.H., Legras, X., Martins, C.S., Wooton-Kee, C.R., et al. (2018). Rapid Disruption of Genes Specifically in Livers of Mice Using Multiplex CRISPR/Cas9 Editing. *Gastroenterology* 155(6)**,** 1967-1970. e1966.

Pliatsika, V., and Rigoutsos, I. (2015). “Off-Spotter”: very fast and exhaustive enumeration of genomic lookalikes for designing CRISPR/Cas guide RNAs. *Biology direct* 10(1)**,** 4.

Poirier, M., Miskel, D., Rings, F., Schellander, K., and Hoelker, M. (2019). 80 Biallelic CRISPR-Cas9 editing of gene associated with coat colour in microinjected bovine zygotes reaching the blastocyst stage. *Reproduction, Fertility and Development* 31(1)**,** 165-165.

Qi, L.S., Larson, M.H., Gilbert, L.A., Doudna, J.A., Weissman, J.S., Arkin, A.P., et al. (2013). Repurposing CRISPR as an RNA-guided platform for sequence-specific control of gene expression. *Cell* 152(5)**,** 1173-1183.

Ramakrishna, S., Dad, A.-B.K., Beloor, J., Gopalappa, R., Lee, S.-K., and Kim, H. (2014). Gene disruption by cell-penetrating peptide-mediated delivery of Cas9 protein and guide RNA. *Genome research*.

Ran, F.A., Cong, L., Yan, W.X., Scott, D.A., Gootenberg, J.S., Kriz, A.J., et al. (2015). In vivo genome editing using Staphylococcus aureus Cas9. *Nature* 520(7546)**,** 186-191.

Ran, F.A., Hsu, P.D., Lin, C.-Y., Gootenberg, J.S., Konermann, S., Trevino, A.E., et al. (2013). Double nicking by RNA-guided CRISPR Cas9 for enhanced genome editing specificity. *Cell* 154(6)**,** 1380-1389.

Roehm, P.C., Shekarabi, M., Wollebo, H.S., Bellizzi, A., He, L., Salkind, J., et al. (2016). Inhibition of HSV-1 replication by gene editing strategy. *Scientific reports* 6**,** 23146.

Ryu, N., Kim, M.-A., Park, D., Lee, B., Kim, Y.-R., Kim, K.-H., et al. (2018). Effective PEI-mediated delivery of CRISPR-Cas9 complex for targeted gene therapy. *Nanomedicine: Nanotechnology, Biology and Medicine* 14(7)**,** 2095-2102.

Shmakov, S., Abudayyeh, O.O., Makarova, K.S., Wolf, Y.I., Gootenberg, J.S., Semenova, E., et al. (2015). Discovery and functional characterization of diverse class 2 CRISPR-Cas systems. *Molecular cell* 60(3)**,** 385-397.

Slaymaker, I.M., Gao, L., Zetsche, B., Scott, D.A., Yan, W.X., and Zhang, F. (2016). Rationally engineered Cas9 nucleases with improved specificity. *Science* 351(6268)**,** 84-88.

Stemmer, M., Thumberger, T., del Sol Keyer, M., Wittbrodt, J., and Mateo, J.L. (2015). CCTop: an intuitive, flexible and reliable CRISPR/Cas9 target prediction tool. *PloS one* 10(4)**,** e0124633.

Strecker, J., Jones, S., Koopal, B., Schmid-Burgk, J., Zetsche, B., Gao, L., et al. (2019). Engineering of CRISPR-Cas12b for human genome editing. *Nature communications* 10(1)**,** 212.

Sun, W., Ji, W., Hall, J.M., Hu, Q., Wang, C., Beisel, C.L., et al. (2015). Self‐assembled DNA nanoclews for the efficient delivery of CRISPR–Cas9 for genome editing. *Angewandte Chemie* 127(41)**,** 12197-12201.

Sun, W., Jiang, T., Lu, Y., Reiff, M., Mo, R., and Gu, Z. (2014). Cocoon-like self-degradable DNA nanoclew for anticancer drug delivery. *Journal of the American Chemical Society* 136(42)**,** 14722-14725.

Tabebordbar, M., Zhu, K., Cheng, J.K., Chew, W.L., Widrick, J.J., Yan, W.X., et al. (2016). In vivo gene editing in dystrophic mouse muscle and muscle stem cells. *Science* 351(6271)**,** 407-411.

Voets, O., Tielen, F., Elstak, E., Benschop, J., Grimbergen, M., Stallen, J., et al. (2017). Highly efficient gene inactivation by adenoviral CRISPR/Cas9 in human primary cells. *PloS one* 12(8)**,** e0182974.

Wang, G., Chow, R.D., Ye, L., Guzman, C.D., Dai, X., Dong, M.B., et al. (2018). Mapping a functional cancer genome atlas of tumor suppressors in mouse liver using AAV-CRISPR–mediated direct in vivo screening. *Science Advances* 4(2)**,** eaao5508.

Watkins-Chow, D.E., Varshney, G.K., Garrett, L.J., Chen, Z., Jimenez, E.A., Rivas, C., et al. (2017). Highly efficient Cpf1-mediated gene targeting in mice following high concentration pronuclear injection. *G3: Genes, Genomes, Genetics* 7(2)**,** 719-722.

Wong, N., Liu, W., and Wang, X. (2015). WU-CRISPR: characteristics of functional guide RNAs for the CRISPR/Cas9 system. *Genome biology* 16(1)**,** 218.

Wright, A.V., Sternberg, S.H., Taylor, D.W., Staahl, B.T., Bardales, J.A., Kornfeld, J.E., et al. (2015). Rational design of a split-Cas9 enzyme complex. *Proceedings of the National Academy of Sciences* 112(10)**,** 2984-2989.

Xu, H., Xiao, T., Chen, C.-H., Li, W., Meyer, C.A., Wu, Q., et al. (2015a). Sequence determinants of improved CRISPR sgRNA design. *Genome research* 25(8)**,** 1147-1157.

Xu, K., Ren, C., Liu, Z., Zhang, T., Zhang, T., Li, D., et al. (2015b). Efficient genome engineering in eukaryotes using Cas9 from Streptococcus thermophilus. *Cellular and molecular life sciences* 72(2)**,** 383-399.

Yamada, M., Watanabe, Y., Gootenberg, J.S., Hirano, H., Ran, F.A., Nakane, T., et al. (2017). Crystal structure of the minimal Cas9 from Campylobacter jejuni reveals the molecular diversity in the CRISPR-Cas9 systems. *Molecular cell* 65(6)**,** 1109-1121. e1103.

Yue, H., Zhou, X., Cheng, M., and Xing, D. (2018). Graphene oxide-mediated Cas9/sgRNA delivery for efficient genome editing. *Nanoscale* 10(3)**,** 1063-1071.

Zetsche, B., Gootenberg, J.S., Abudayyeh, O.O., Slaymaker, I.M., Makarova, K.S., Essletzbichler, P., et al. (2015). Cpf1 is a single RNA-guided endonuclease of a class 2 CRISPR-Cas system. *Cell* 163(3)**,** 759-771.

Zhang, R., Miner, J.J., Gorman, M.J., Rausch, K., Ramage, H., White, J.P., et al. (2016). A CRISPR screen defines a signal peptide processing pathway required by flaviviruses. *Nature* 535(7610)**,** 164.

Zhu, L.J., Holmes, B.R., Aronin, N., and Brodsky, M.H. (2014). CRISPRseek: a bioconductor package to identify target-specific guide RNAs for CRISPR-Cas9 genome-editing systems. *PloS one* 9(9)**,** e108424.
